# Non-invasive brain stimulation for cognitive impairment after traumatic brain injury: an umbrella review of systematic reviews

**DOI:** 10.3389/fneur.2026.1931683

**Published:** 2026-08-19

**Authors:** Shouli Li, Chongyue Liu, Jinyue Li, Yuemu Li

**Affiliations:** Heilongjiang Academy of Chinese Medicine Sciences, Harbin, China

**Keywords:** AMSTAR-2, cognition, non-invasive brain stimulation, repetitive transcranial magnetic stimulation, transcranial direct current stimulation, traumatic brain injury, umbrella review

## Abstract

**Background:**

Cognitive impairment is a frequent and disabling consequence of traumatic brain injury (TBI). Non-invasive brain stimulation (NIBS), including repetitive transcranial magnetic stimulation (rTMS) and transcranial direct current stimulation (tDCS), has been investigated as a potential adjunctive intervention, but review-level conclusions remain inconsistent.

**Methods:**

We conducted an umbrella review of published systematic reviews evaluating NIBS for cognitive outcomes after TBI. PubMed, Cochrane Library, Embase, Web of Science, CNKI, and Wanfang were searched from inception to May 2026. The unit of analysis was the systematic review, not individual primary trials. Eligible reviews assessed rTMS, tDCS, or both, and reported cognitive outcomes. Methodological quality was reappraised using AMSTAR-2, and primary-study overlap was assessed from the existing review-by-study citation matrix using corrected covered area (CCA). Where reviews reported quantitative estimates for cognition, these estimates were summarized descriptively at the review level; no *de novo* primary-study meta-analysis and no independent treatment-effect estimation were performed. The protocol was registered in PROSPERO (CRD420261395308).

**Results:**

Twenty-nine records were identified. Ten review records were included, consisting of seven core evidence reviews and three supplementary reviews. Eight reviews directly addressed cognitive outcomes, while supplementary reviews provided contextual review-level evidence. Conservative AMSTAR-2 reassessment found no high- or moderate-quality reviews; two were rated low and eight critically low in the complete 10-record assessment. The citation matrix contained 42 unique primary studies and 92 primary-study occurrences across 10 review records, yielding a CCA of 13.23%, consistent with high overlap. Previously published quantitative review-level estimates showed inconsistent directions of evidence and did not demonstrate consistent evidence of an overall cognitive benefit.

**Conclusion:**

Current review-level evidence does not demonstrate consistent evidence of an overall cognitive benefit of NIBS after TBI. Because available reviews are methodologically limited and clinically heterogeneous, routine use of NIBS for post-TBI cognitive rehabilitation is not supported outside research or carefully selected clinical contexts.

## Introduction

Traumatic brain injury (TBI) is a major cause of long-term neurological disability. Cognitive impairment after TBI commonly affects attention, memory, processing speed, executive function, and social participation, and may persist beyond the acute recovery period. These deficits reduce independence, interfere with return to work, and increase the burden on families and health-care systems.

Conventional cognitive rehabilitation, pharmacological strategies, and multidisciplinary neurorehabilitation are used in practice, but treatment effects are variable. Non-invasive brain stimulation (NIBS), including rTMS and tDCS, may modulate cortical excitability and network plasticity and has therefore been investigated as an adjunctive approach for cognitive impairment after TBI.

Existing systematic reviews have reported mixed conclusions, partly because primary studies differ in injury severity, chronicity, stimulation target, stimulation parameters, comparator condition, and cognitive outcome measures. An umbrella review can clarify the review-level evidence, examine methodological quality, and identify whether apparent improvement signals are robust enough to support clinical translation.

## Methods

### Protocol and reporting

This umbrella review followed PRISMA 2020 where applicable and reporting guidance for overviews of reviews, including the PRIOR framework. The review protocol was prospectively registered in PROSPERO (CRD420261395308) on 14 May 2026, before completion of review selection and data extraction. No major deviations from the registered protocol occurred. Minor reporting clarifications were made regarding eligibility definitions, core/supplementary evidence classification, and overlap assessment. No deviation involved adding a new primary-study meta-analysis or changing the review-level unit of analysis.

### Umbrella review design and unit of analysis

The review was designed as an umbrella review, also referred to as an overview of reviews. Accordingly, the unit of analysis was the published systematic review rather than the individual randomized or non-randomized primary trial. Primary studies were considered only to describe the evidence base represented within the included reviews, to identify duplicate citation of the same primary studies across reviews, and to interpret whether review-level findings were independent. Ten review records were included in the umbrella evidence map, consisting of seven core evidence reviews and three supplementary reviews. Eight reviews directly addressed cognitive outcomes, while supplementary reviews provided contextual evidence. We did not extract patient-level data, recalculate primary-study estimates, conduct a *de novo* primary-study meta-analysis, or generate an independent combined estimate of clinical efficacy. Quantitative estimates reported by the included reviews were interpreted descriptively as review-level evidence.

### Search strategy

PubMed, Cochrane Library, Embase, Web of Science, CNKI, and Wanfang were searched from inception to May 2026. An updated search close to resubmission was not performed because this revision was limited to methodological clarification, reporting improvement, and consistency checks rather than evidence updating. Accordingly, the potential impact of review-level evidence published after May 2026 is acknowledged as a limitation. Search terms combined concepts for traumatic brain injury, NIBS modalities, cognition, and systematic reviews/meta-analyses. Searches were designed to identify review-level evidence rather than individual primary trials. No additional language or publication-status restrictions were specified beyond database coverage and report retrievability. No numerical database-specific yield was inferred beyond the documented total records because the available screening file reported only aggregate counts. Database-specific strategies are provided in the Supplementary materials. Reference lists of included reviews were checked for potentially relevant review-level records when available; no trial-registry search was used to identify primary trials because primary studies were not the unit of analysis.

The core PubMed strategy was: ((“Brain Injuries, Traumatic”[Mesh] OR “traumatic brain injury” OR TBI OR “head injury” OR concussion OR “brain trauma”) AND (“Transcranial Magnetic Stimulation”[Mesh] OR “transcranial magnetic stimulation” OR rTMS OR “theta burst stimulation” OR “transcranial direct current stimulation” OR tDCS OR “non-invasive brain stimulation” OR “noninvasive brain stimulation”) AND (cognit* OR attention OR memory OR “executive function” OR “processing speed” OR “verbal fluency” OR neuropsycholog*) AND (“systematic review” OR meta-analysis OR metaanalysis OR review)). Equivalent controlled-vocabulary and free-text strategies were adapted for Embase, Cochrane Library, Web of Science, CNKI, and Wanfang. Chinese database terms included traumatic brain injury/brain trauma, transcranial magnetic stimulation, transcranial direct current stimulation, non-invasive brain stimulation, cognition, systematic review, and meta-analysis.

### Eligibility criteria

Eligibility criteria followed the PICOS framework and were operationalized at the review level. Eligible populations were patients with traumatic brain injury, including mild, moderate, severe, or mixed-severity TBI, only when the review reported TBI-specific data or when the TBI subgroup was clearly identifiable. Reviews in which TBI data could not be separated from other neurological or psychiatric populations were not treated as core cognitive evidence. Eligible interventions were non-invasive brain stimulation modalities relevant to cognitive rehabilitation after TBI, including rTMS, tDCS, or mixed NIBS approaches; reviews of combined NIBS plus cognitive training were eligible when the stimulation component and cognitive outcomes were clearly described. Cognitive outcomes were defined as global cognitive function or specific domains such as memory, attention, processing speed, executive function, working memory, verbal fluency, or cognition-related neuropsychological measures. Physiological measures such as P300 were considered cognition-related only when the original review linked them to cognitive assessment or rehabilitation. Comparators were sham stimulation, conventional rehabilitation, usual care, cognitive training, or other control conditions as defined by the included reviews. Eligible study designs were systematic reviews with or without meta-analysis. Reviews without cognitive or cognition-related outcomes, articles that were not systematic reviews, and reports from which TBI-specific cognitive evidence could not be extracted were excluded.

### Study selection and data extraction

Two reviewers independently screened titles and abstracts against the predefined review-level eligibility criteria. Potentially eligible reports were then assessed in full text by two reviewers independently. Disagreements at either screening stage were resolved through discussion; when consensus could not be reached, a third reviewer adjudicated. Data extraction was performed independently using a standardized extraction form and then cross-checked for accuracy. Extracted variables included review identification details, population characteristics, TBI severity, TBI chronicity or time since injury when reported, NIBS modality, stimulation target and parameters, number of sessions, comparator, cognitive domains and outcome measures, review-reported quantitative estimates when available, adverse events or safety reporting, risk-of-bias tool used by the review, and availability of GRADE or other certainty assessments. Primary-study lists used for overlap assessment were extracted at the review level, deduplicated by study author, year, title, DOI, and population/intervention details, and verified by reviewer consensus.

#### Core and supplementary evidence classification

Reviews were classified using predefined review-level decision rules that were independent of the direction of findings. Core evidence reviews were systematic reviews focused on NIBS for cognitive or cognition-related outcomes in TBI populations, with TBI-specific cognitive evidence extractable from the report. Supplementary evidence reviews were broader-scope reviews that included relevant TBI/NIBS/cognition information but did not focus primarily on post-TBI cognitive rehabilitation, included mixed neuropsychiatric or broader rehabilitation populations, or addressed cognition as a secondary or contextual outcome. Supplementary reviews were used to contextualize the evidence base and overlap structure, but they were not treated as independent confirmation of benefit.

### Methodological quality, evidence certainty, and primary-study overlap

Methodological quality of the included systematic reviews was assessed using AMSTAR-2, which evaluates the conduct and reporting of systematic reviews across 16 methodological domains. AMSTAR-2 confidence ratings were interpreted as review-level methodological quality, not as direct certainty of treatment effects. Overall confidence ratings were reassigned conservatively from the complete item-level table: reviews with more than one critical weakness were rated critically low, and reviews with one critical weakness were rated low. Critical domains considered in interpretation included protocol registration, comprehensiveness of search, excluded-study lists, risk-of-bias assessment, appropriateness of statistical combination in the original review, consideration of risk of bias in interpretation, and assessment or discussion of publication bias when an included review statistically combined findings. GRADE, when reported by the original reviews, was extracted separately because it evaluates outcome-level certainty based on risk of bias, inconsistency, indirectness, imprecision, and publication bias. Because GRADE frameworks, outcomes, and populations differed across included reviews, we did not assign a new cross-review GRADE rating.

Primary-study overlap was assessed using the existing unique-primary-study list and review-by-study citation matrix. Each row of the matrix represented a unique primary study and each column represented an included review record. Corrected covered area (CCA) was calculated as CCA = (*N* − *r*)/(r*c − *r*), where *N* is the total number of primary-study occurrences across reviews, r is the number of unique primary studies, and c is the number of reviews in the citation matrix. The citation matrix, pairwise sharing patterns, and the most frequently repeated primary studies were used to judge the independence of review-level evidence. Overlap information was incorporated into interpretation by avoiding numerical vote-counting across reviews and by not treating concordant conclusions from highly overlapping reviews as independent confirmations of effect.

### Descriptive presentation of previously reported review-level estimates

Where included reviews reported quantitative estimates for cognition, we presented these previously published estimates descriptively. This descriptive presentation was used only to display the direction and magnitude of previously reported review-level estimates and was not intended as a combined analysis. We did not apply a *de novo* primary-study meta-analysis, did not recalculate primary-study estimates, and did not treat overlapping review records as independent primary evidence. Because Galimberti et al. (1) reported a standardized mean change rather than a conventional between-group SMD, all estimates were interpreted only as review-level findings reported by the original reviews.

## Results

### Study selection

**Figure 1:**
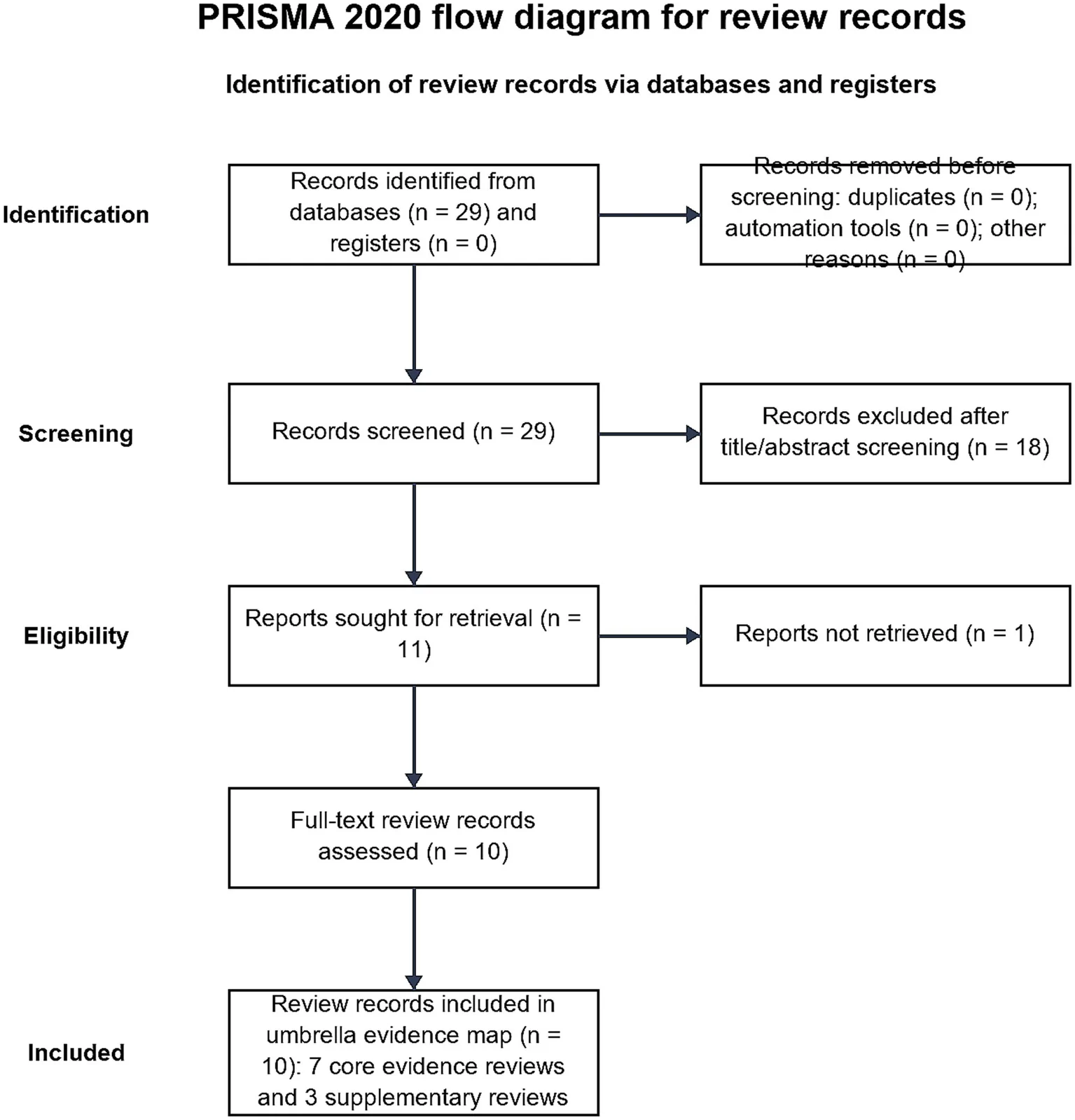
PRISMA 2020 flow diagram showing record identification, screening, eligibility assessment, and inclusion.

The search identified 29 records. No duplicates were removed. Twenty-nine records were screened by title and abstract, and 18 were excluded. Eleven reports were sought for retrieval, of which one was excluded because the full text remained unavailable after manual verification. Ten full-text review records were assessed and included in the umbrella evidence map. Overall, the evidence base consisted of seven core evidence reviews and three supplementary reviews. Eight reviews directly addressed cognitive outcomes, while supplementary reviews provided contextual review-level evidence (Figure 1).

### Characteristics of included reviews

Ten review records were included, consisting of seven core evidence reviews and three supplementary reviews. Eight reviews directly addressed cognitive outcomes, while supplementary reviews provided contextual evidence. NIBS modalities included rTMS alone, tDCS alone, rTMS combined with cognitive training, or mixed rTMS and tDCS approaches. Reviews included 5–44 primary studies overall, although several broad reviews included studies outside the cognitive NIBS question and were interpreted only for their relevant review-level findings (see Table 1).

**Table 1:** Characteristics of included review records.

Author, year	Type	NIBS	Population	Studies/participants	Cognitive outcomes	Review-level interpretation	Set
Ahorsu et al. (2021) (2)	SR + MA	Mixed NIBS	TBI; severity not restricted	9; 197	overall/global cognition; attention; memory; executive function	Reported estimates suggested possible benefit, but confidence in the evidence remained limited by sample size, heterogeneity, and methodological concerns.	Core
Alashram et al. (2023) (6)	SR	rTMS/tDCS	Mixed TBI severity/stage	10; 228	Global cognition; memory; attention; EF	Narrative evidence was limited and heterogeneous.	Core
Aleid et al. (2025) (3)	SR + MA of RCTs	rTMS	TBI; severity NR	7; 184	Cognitive function	Reported estimates suggested possible benefit, but confidence in the evidence remained limited by sparse domain detail and methodological concerns.	Core
Chen (2024) (9)	SR + MA	rTMS + cognitive training	Post-TBI cognitive disorder	14; 820	MoCA; MMSE; memory; attention; P300	Reported improvement signals for combined rTMS + cognitive training, although interpretation was limited by heterogeneity and methodological concerns; evidence did not isolate rTMS alone.	Core
Nousia et al. (2022) (5)	SR	NIBS +/− cognitive training	Mixed mild/moderate/severe TBI	10; 232	Attention; EF; speed; WM; memory	No clear support for NIBS or NIBS-CT improving cognition.	Core
Hara et al. (2021) (7)	SR	rTMS/tDCS	TBI rehabilitation patients	5; 135	Attention; memory; WM; EF	Preliminary mixed findings; insufficient evidence for routine effectiveness.	Core
Tsai et al. (2021) (4)	SR + MA	rTMS	TBI with depression/headache/PCS	7; 136	Visuospatial memory; speed; attention	Reported improvement signals for visuospatial memory, although interpretation was limited; other cognitive domains were not consistently demonstrated.	Core
Kawata (2024) (10)	SR of RCTs	Stimulation/NIBS findings only	Adults with chronic TBI; broad review	44 RCTs overall; participants NR	Cognition; WM; speed; fatigue	Broad review; stimulation/NIBS cognition findings only.	Supplementary
Zaninotto et al. (2019) (8)	SR	tDCS	Adult TBI; mixed recovery outcomes	14; NR	Cognition; neuropsychology; DOC/motor	Broad tDCS review with mixed cognition, motor, and DOC outcomes.	Supplementary
Oktaviani (2026) (11)	MA + TSA	rTMS	TBI; pain/neuropsychiatric focus	9; NR	Cognitive function; PCS; depression; pain	Supplementary/sensitivity evidence; cognition secondary.	Supplementary

EF, executive function; MMSE, Mini-Mental State Examination; MoCA, Montreal Cognitive Assessment; NIBS, non-invasive brain stimulation; NR, not reported; RCT, randomized controlled trial; rTMS, repetitive transcranial magnetic stimulation; SR, systematic review; tDCS, transcranial direct current stimulation; TBI, traumatic brain injury; WM, working memory.

### Core evidence sensitivity interpretation

Restricting interpretation to the seven core evidence reviews did not change the umbrella-review conclusions. The core reviews remained methodologically limited and clinically heterogeneous, and their review-level findings did not provide consistent evidence of an overall cognitive benefit. The three supplementary reviews were used only as contextual review-level evidence and did not drive the main conclusions.

### Methodological quality

**Figure 2:**
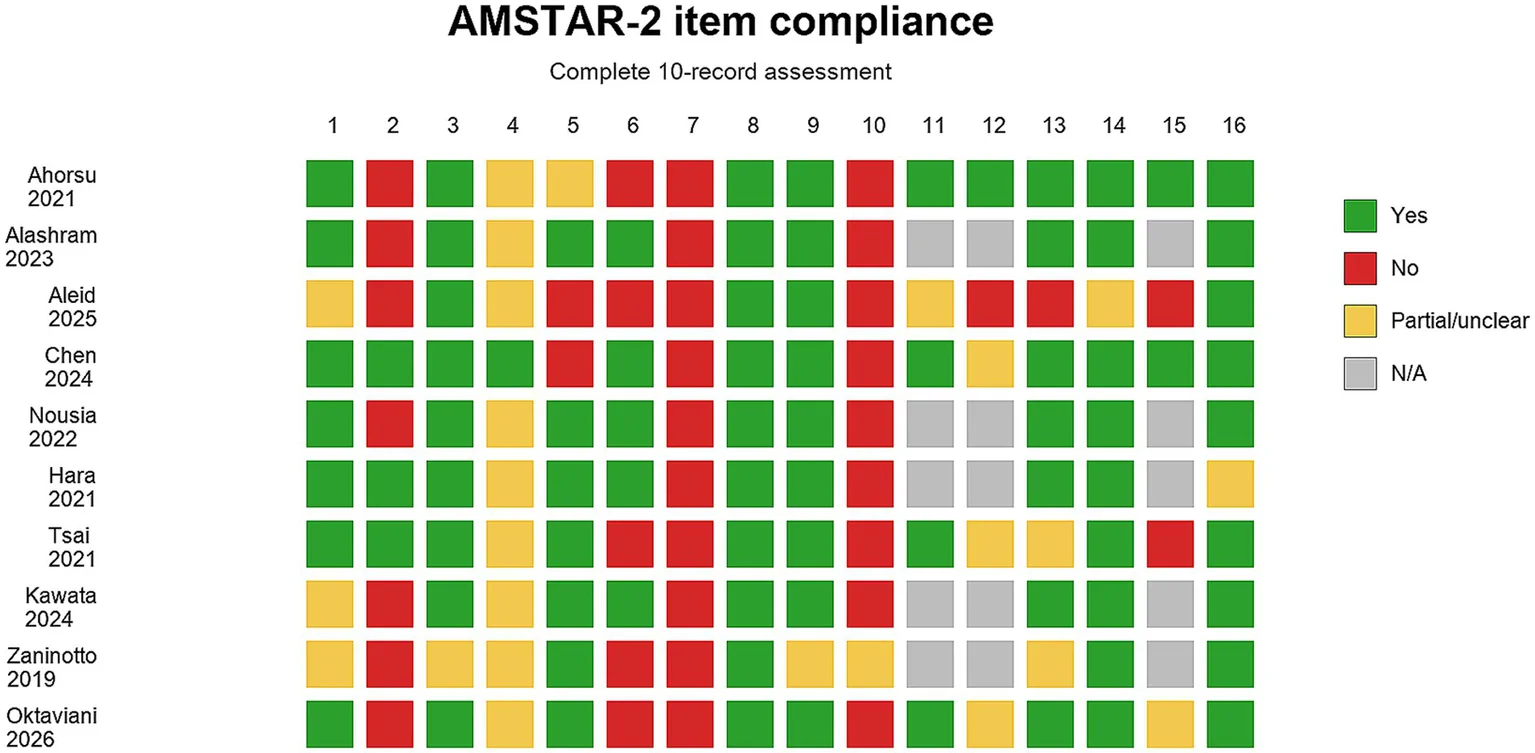
AMSTAR-2 methodological quality heat map based on the complete item-level assessment. Green indicates yes, red no, yellow unclear, and gray not applicable. Items 1–16 correspond to AMSTAR-2 domains. Ratings were assigned conservatively; no numerical total score was calculated.

AMSTAR-2 item-level judgments are shown in Figure 2 and the complete assessment is provided in the Supplementary materials. Conservative reassessment of the complete 10-record set found no high- or moderate-quality reviews. Two reviews were rated low quality and eight critically low quality. The most frequent critical weaknesses were lack of protocol registration and absence of excluded-study lists. In quantitative reviews, assessment or discussion of publication bias and incorporation of risk of bias into interpretation were incomplete in some cases. These weaknesses substantially reduce confidence in the review-level conclusions and support cautious interpretation of apparent improvement signals.

### Primary-study overlap

The overlap matrix identified 42 unique primary studies and 92 primary-study occurrences across 10 review records. The resulting CCA was 13.23%, indicating high primary-study overlap. The most frequently repeated primary study was Lee (12), which appeared in nine review records. Several review pairs shared six unique primary studies. These findings indicate that review-level conclusions were not fully independent and should not be interpreted as separate confirmations of effect.

### Descriptive presentation of previously reported review-level estimates

**Figure 3:**
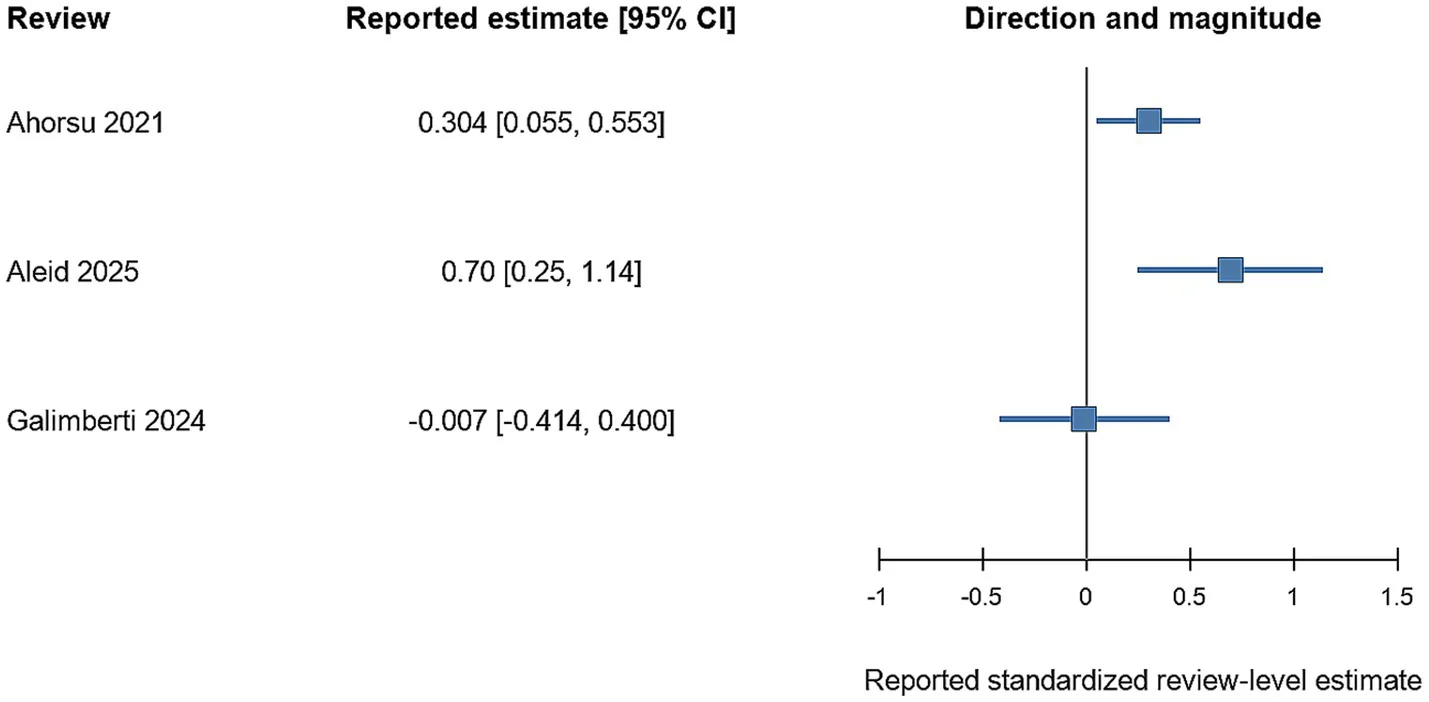
Previously reported estimates extracted from included reviews (descriptive presentation only).

Three previously published reviews reported quantitative estimates for overall cognition that could be displayed descriptively: Ahorsu et al. (2), Aleid et al. (3), and Galimberti et al. (1). The previously reported review-level estimates showed inconsistent directions of evidence, and the available evidence did not demonstrate a consistent overall cognitive benefit. This descriptive presentation was used only to display the direction and magnitude of previously reported review-level estimates and was not intended as a combined analysis. The visual representation is shown in Figure 3.

### Cognitive domain-specific findings

Domain-specific evidence was sparse and inconsistent. Tsai et al. (4) reported a review-level improvement signal for visuospatial memory after rTMS, whereas findings for processing speed and selective attention were not consistently demonstrated. Nousia et al. (5) did not report consistent review-level support for NIBS combined with cognitive training over cognitive training alone. Across the included reviews, outcome measures, stimulation protocols, TBI populations, and follow-up durations varied substantially, limiting certainty and generalizability.

### Safety reporting

Adverse-event reporting was limited and inconsistently presented across the included review records. Reported adverse events in individual reviews, when available, included headache, scalp discomfort, and treatment discontinuation; however, modality-specific safety reporting was insufficient across the included reviews. Some reviews focused primarily on cognitive outcomes and did not provide detailed, outcome-level safety summaries for the TBI cognitive rehabilitation population. Consequently, the available review-level evidence was insufficient to draw firm conclusions about tolerability or comparative safety. Incomplete reporting of adverse events should not be interpreted as confirming safety.

## Discussion

This umbrella review synthesized 10 published review records evaluating NIBS-related evidence for cognitive impairment after TBI, including seven core evidence reviews and three supplementary reviews. Eight reviews directly addressed cognitive outcomes, while supplementary reviews provided contextual review-level evidence. The purpose was not to independently re-estimate the treatment effect of NIBS, but to summarize existing systematic reviews, appraise review quality, assess primary-study overlap, and judge the reliability and consistency of current review-level evidence. Previously published review-level evidence remains uncertain: some reviews reported improvement signals, but the review-level estimates were inconsistent, and methodological confidence was low or critically low across the assessed reviews.

The evidence appraisal helps explain why reported improvement signals from individual reviews should be interpreted cautiously. Ahorsu et al. (2) and Aleid et al. (3) reported possible improvement signals or positive estimates at the review level, whereas Galimberti et al. (1) found no clear review-level support. These differences may reflect variation in eligibility criteria, intervention modality, outcome definitions, and reported metrics rather than true independent replication. Because primary-study overlap was high, repeated citation of the same trials across reviews may amplify apparent consistency if overlap is not explicitly considered.

Clinical and methodological heterogeneity likely contributed to inconsistent findings. Stimulation protocols varied by modality, target, frequency, intensity, session number, treatment duration, and whether stimulation was combined with cognitive training or rehabilitation. TBI populations differed in severity, chronicity, and baseline cognitive impairment. Cognitive outcome measures also varied substantially, limiting comparability across reviews and domains.

The AMSTAR-2 reassessment has direct implications for interpretation. The absence of protocol registration and excluded-study lists weakens transparency and increases the possibility of selective methods. Incomplete reporting of publication-bias assessment, unclear duplicate extraction in some reviews, and limited integration of risk of bias into interpretation further reduce confidence. Because umbrella reviews depend on the methodological reliability of included reviews, these issues limit the strength of any clinical recommendation. Where evidence certainty was addressed by the original reviews, approaches were inconsistent; therefore, certainty was considered narratively rather than converted into a new cross-review certainty rating. Accordingly, the conclusions of this umbrella review should be interpreted as an appraisal of the reliability and consistency of existing review-level evidence rather than an independent estimate of treatment efficacy.

This review has limitations. The evidence base is small, clinically heterogeneous, and affected by high primary-study overlap. Only three reviews reported quantitative estimates for overall cognition that could be displayed descriptively. These estimates used different standardized metrics reported by the original reviews; therefore, they were treated as contextual review-level evidence rather than as inputs for an independent combined estimate of clinical efficacy. Publication bias could not be meaningfully assessed at the umbrella-review level because of the small number of reported estimates. Aggregate review-level data did not allow patient-level or stimulation-parameter subgroup analyses.

Clinically, the available evidence does not justify routine use of NIBS as a standard cognitive rehabilitation intervention after TBI. NIBS may remain appropriate in research settings or carefully selected clinical contexts, but current review-level evidence is insufficient to establish durable, generalizable cognitive benefit. Any clinical application should emphasize appropriate patient selection, standardized stimulation parameters, and meaningful cognitive outcomes. Future RCTs should use preregistered protocols, sham-controlled double-blind designs, adequate sample sizes, standardized cognitive outcome batteries, clear TBI phenotyping, and follow-up assessments. Future systematic reviews should preregister protocols, provide excluded-study lists, use duplicate screening and extraction, assess publication bias when appropriate, report certainty of evidence by outcome, and account for primary-study overlap.

## Conclusion

Current review-level evidence does not demonstrate consistent evidence of an overall cognitive benefit of NIBS for patients with TBI. The evidence base is constrained by low methodological quality, high primary-study overlap, clinical heterogeneity, and imprecision. NIBS should therefore be considered investigational or selectively applied rather than established as routine cognitive rehabilitation after TBI.

## Data Availability

The original contributions presented in the study are included in the article/Supplementary material, further inquiries can be directed to the corresponding author.
