# Supplementary material for: Non-invasive brain stimulation for cognitive impairment after traumatic brain injury: an umbrella review of systematic reviews
Source: Front Neurol. 2026 Aug 19;17:1931683. doi: 10.3389/fneur.2026.1931683 (PMC13533710; doi:10.3389/fneur.2026.1931683)
Supplement: Supplementary file 1 [file Table_1.DOCX]

Supplementary Table S1. Search strategies.

| Database | Search strategy |
| --- | --- |
| PubMed | ("Brain Injuries, Traumatic"[Mesh] OR "traumatic brain injury" OR TBI OR "head injury" OR concussion OR "brain trauma") AND ("Transcranial Magnetic Stimulation"[Mesh] OR "transcranial magnetic stimulation" OR rTMS OR "theta burst stimulation" OR "transcranial direct current stimulation" OR tDCS OR "non-invasive brain stimulation" OR "noninvasive brain stimulation") AND (cognit* OR attention OR memory OR "executive function" OR "processing speed" OR "verbal fluency" OR neuropsycholog*) AND ("systematic review" OR meta-analysis OR metaanalysis OR review) |
| Embase | ('traumatic brain injury'/exp OR 'traumatic brain injury' OR tbi OR 'head injury' OR concussion) AND ('transcranial magnetic stimulation'/exp OR rtms OR 'theta burst stimulation' OR 'transcranial direct current stimulation' OR tdcs OR 'non-invasive brain stimulation') AND (cognit* OR attention OR memory OR 'executive function' OR 'processing speed' OR neuropsycholog*) AND ('systematic review'/exp OR 'meta analysis'/exp OR 'systematic review' OR meta-analysis) |
| Cochrane Library | ("traumatic brain injury" OR TBI OR "head injury" OR concussion) AND ("transcranial magnetic stimulation" OR rTMS OR "transcranial direct current stimulation" OR tDCS OR "non-invasive brain stimulation") AND (cognition OR cognitive OR attention OR memory OR executive) |
| Web of Science | TS=(("traumatic brain injury" OR TBI OR "head injury" OR concussion) AND ("transcranial magnetic stimulation" OR rTMS OR "transcranial direct current stimulation" OR tDCS OR "non-invasive brain stimulation") AND (cognit* OR attention OR memory OR "executive function" OR neuropsycholog*) AND ("systematic review" OR meta-analysis OR review)) |
| CNKI | 主题=("创伤性脑损伤" OR "颅脑损伤" OR "脑外伤") AND 主题=("经颅磁刺激" OR "重复经颅磁刺激" OR "经颅直流电刺激" OR "无创脑刺激") AND 主题=("认知" OR "注意" OR "记忆" OR "执行功能") AND 主题=("系统评价" OR "Meta分析" OR "荟萃分析") |
| Wanfang | 主题或关键词=("创伤性脑损伤" OR "颅脑损伤" OR "脑外伤") AND ("经颅磁刺激" OR "重复经颅磁刺激" OR "经颅直流电刺激" OR "无创脑刺激") AND ("认知" OR "注意" OR "记忆" OR "执行功能") AND ("系统评价" OR "Meta分析" OR "荟萃分析") |
