# Supplementary material for: Non-invasive brain stimulation for cognitive impairment after traumatic brain injury: an umbrella review of systematic reviews
Source: Front Neurol. 2026 Aug 19;17:1931683. doi: 10.3389/fneur.2026.1931683 (PMC13533710; doi:10.3389/fneur.2026.1931683)
Supplement: Supplementary file 2 [file Table_2.DOCX]

**Supplementary Table S2. AMSTAR-2 assessment.**

Each AMSTAR-2 item was rated as Yes, Partial Yes, No, or Not applicable. No numerical total score was calculated.

| **Review ID** | **Review** | **Overall confidence** | **Critical weaknesses** | **Non-critical weaknesses** | **Notes** |
| --- | --- | --- | --- | --- | --- |
| R01 | Ahorsu 2021 | Critically low | Q2, Q7 | Q4 partial, Q5 partial, Q6, Q10 | Meta-analysis; rating driven down mainly by no prospective registration and no excluded-study list. |
| R02 | Alashram 2023 | Critically low | Q2, Q7 | Q4 partial, Q10 | Narrative systematic review; no meta-analysis. |
| R03 | Aleid 2025 | Critically low | Q2, Q7, Q13, Q15 | Q1 partial, Q4 partial, Q5, Q6, Q10, Q11 partial, Q12, Q14 partial | Meta-analysis; several core AMSTAR-2 safeguards were not reported. |
| R04 | Chen 2024 | Low | Q7 | Q5, Q10, Q12 partial | Meta-analysis of rTMS plus cognitive training; effects should not be attributed to rTMS alone. |
| R05 | Nousia 2022 | Critically low | Q2, Q7 | Q4 partial, Q10 | Narrative systematic review; conclusions appropriately cautious. |
| R06 | Hara 2021 | Low | Q7 | Q4 partial, Q10, Q16 partial | Narrative systematic review with prospective PROSPERO registration. |
| R07 | Tsai 2021 | Critically low | Q7, Q15 | Q4 partial, Q6, Q10, Q12 partial, Q13 partial | Meta-analysis; cognition was a secondary aim alongside depression/PCS. |
| R08 | Kawata 2024 | Critically low | Q2, Q7 | Q1 partial, Q4 partial, Q10 | Broad-scope RCT review; only stimulation/NIBS cognition-relevant findings should inform this umbrella review. |
| R09 | Zaninotto 2019 | Critically low | Q2, Q7 | Q1 partial, Q3 partial, Q4 partial, Q6, Q9 partial, Q10 partial, Q13 partial | Broad outcome review including cognition, motor and DOC/consciousness outcomes. |
| R10 | Oktaviani 2026 | Critically low | Q2, Q7 | Q4 partial, Q6, Q10, Q12 partial, Q15 partial | Supplementary/sensitivity evidence; primary focus was pain/neuropsychiatric outcomes, not cognition. |

| **Review ID** | **Review** | **Item** | **Domain** | **Rating** | **Justification** |
| --- | --- | --- | --- | --- | --- |
| R01 | Ahorsu 2021 | Q1 | PICO components | Yes | PICO eligibility criteria explicitly defined participants with TBI, NIBS intervention, sham/control comparison, and cognitive outcomes. |
| R01 | Ahorsu 2021 | Q2 | protocol registered before review | No | The review stated that methods followed PRISMA-P but were not prospectively registered. |
| R01 | Ahorsu 2021 | Q3 | explanation of study designs | Yes | Eligible designs and JBI tools for quasi-experimental and randomized controlled studies were specified. |
| R01 | Ahorsu 2021 | Q4 | comprehensive search strategy | Partial Yes | Five databases and keywords were reported, with English restriction; grey literature and full database-specific strategies were not fully documented in the main text. |
| R01 | Ahorsu 2021 | Q5 | study selection duplicate | Partial Yes | Literature search and study selection were performed by two named reviewers with supervision, but independent duplicate selection was not explicitly stated. |
| R01 | Ahorsu 2021 | Q6 | data extraction duplicate | No | Duplicate or checked data extraction was not clearly reported. |
| R01 | Ahorsu 2021 | Q7 | excluded studies list | No | A PRISMA flow and exclusion counts were provided, but no list of excluded full-text studies with reasons was reported. |
| R01 | Ahorsu 2021 | Q8 | included studies description | Yes | Included studies were summarized in tables with participants, interventions, outcomes, quality and risk-of-bias information. |
| R01 | Ahorsu 2021 | Q9 | risk of bias assessment | Yes | JBI critical appraisal and Cochrane risk-of-bias domains were used. |
| R01 | Ahorsu 2021 | Q10 | funding sources of primary studies | No | Funding sources of the included primary studies were not reported. |
| R01 | Ahorsu 2021 | Q11 | meta analysis methods | Yes | Random-effects meta-analysis, standardized mean differences, heterogeneity statistics, meta-regression and sensitivity analyses were described. |
| R01 | Ahorsu 2021 | Q12 | impact of RoB on results | Yes | Sensitivity analyses considered study design and risk of bias. |
| R01 | Ahorsu 2021 | Q13 | RoB considered in interpretation | Yes | Interpretation referred to quality and risk of bias of included studies. |
| R01 | Ahorsu 2021 | Q14 | heterogeneity explained | Yes | Heterogeneity was quantified and potential moderators were explored. |
| R01 | Ahorsu 2021 | Q15 | publication bias | Yes | Funnel plots were used to assess publication bias. |
| R01 | Ahorsu 2021 | Q16 | conflict of interest | Yes | The review reported no external funding and no conflicts of interest. |
| R02 | Alashram 2023 | Q1 | PICO components | Yes | PICOS eligibility criteria specified TBI population, rTMS/tDCS interventions, control groups, cognitive outcomes, and study designs. |
| R02 | Alashram 2023 | Q2 | protocol registered before review | No | No protocol registration or prospective protocol was reported. |
| R02 | Alashram 2023 | Q3 | explanation of study designs | Yes | RCTs, controlled clinical trials and pilot studies were eligible and justified by the review question. |
| R02 | Alashram 2023 | Q4 | comprehensive search strategy | Partial Yes | Seven databases, search terms and PRISMA reporting were described, but restrictions to English and lack of grey-literature searching limited comprehensiveness. |
| R02 | Alashram 2023 | Q5 | study selection duplicate | Yes | Two authors independently screened titles/abstracts and full texts, with disagreements resolved by a third author. |
| R02 | Alashram 2023 | Q6 | data extraction duplicate | Yes | One author extracted data and another checked the extraction. |
| R02 | Alashram 2023 | Q7 | excluded studies list | No | The review gave flow counts but did not provide a list of excluded full-text studies with reasons. |
| R02 | Alashram 2023 | Q8 | included studies description | Yes | Tables reported study design, sample size, intervention characteristics, outcomes and methodological quality. |
| R02 | Alashram 2023 | Q9 | risk of bias assessment | Yes | Cochrane risk-of-bias instrument and PEDro scale were used as appropriate. |
| R02 | Alashram 2023 | Q10 | funding sources of primary studies | No | Funding sources of primary studies were not reported. |
| R02 | Alashram 2023 | Q11 | meta analysis methods | Not Applicable | No meta-analysis was conducted because of heterogeneity in outcomes and protocols. |
| R02 | Alashram 2023 | Q12 | impact of RoB on results | Not Applicable | No meta-analysis was conducted. |
| R02 | Alashram 2023 | Q13 | RoB considered in interpretation | Yes | The discussion emphasized limited evidence, low methodological quality and small samples. |
| R02 | Alashram 2023 | Q14 | heterogeneity explained | Yes | The review discussed heterogeneity in protocols, injury duration and outcomes. |
| R02 | Alashram 2023 | Q15 | publication bias | Not Applicable | No meta-analysis was conducted. |
| R02 | Alashram 2023 | Q16 | conflict of interest | Yes | The authors reported no conflict of interest and no funding. |
| R03 | Aleid 2025 | Q1 | PICO components | Partial Yes | The review specified RCTs of rTMS in TBI and outcomes, but PICO elements were only briefly described. |
| R03 | Aleid 2025 | Q2 | protocol registered before review | No | No protocol registration or prior protocol was reported. |
| R03 | Aleid 2025 | Q3 | explanation of study designs | Yes | The review restricted inclusion to randomized controlled trials of rTMS in TBI. |
| R03 | Aleid 2025 | Q4 | comprehensive search strategy | Partial Yes | Three databases and two main keywords were reported; search breadth, grey literature and full strategies were limited. |
| R03 | Aleid 2025 | Q5 | study selection duplicate | No | Duplicate independent study selection was not reported. |
| R03 | Aleid 2025 | Q6 | data extraction duplicate | No | Duplicate or checked data extraction was not reported. |
| R03 | Aleid 2025 | Q7 | excluded studies list | No | Flow counts and reasons categories were reported, but no excluded full-text list was provided. |
| R03 | Aleid 2025 | Q8 | included studies description | Yes | Baseline characteristics and included RCTs were tabulated. |
| R03 | Aleid 2025 | Q9 | risk of bias assessment | Yes | RoB 2 was used to assess randomized trials. |
| R03 | Aleid 2025 | Q10 | funding sources of primary studies | No | Funding sources of the included primary studies were not reported. |
| R03 | Aleid 2025 | Q11 | meta analysis methods | Partial Yes | RevMan, SMDs, fixed/random models and I2 were described, but methods were sparsely reported. |
| R03 | Aleid 2025 | Q12 | impact of RoB on results | No | The influence of risk of bias on pooled results was not clearly examined. |
| R03 | Aleid 2025 | Q13 | RoB considered in interpretation | No | Risk-of-bias findings were reported but not clearly incorporated into interpretation of the evidence. |
| R03 | Aleid 2025 | Q14 | heterogeneity explained | Partial Yes | I2 was reported, but exploration of sources of heterogeneity was limited. |
| R03 | Aleid 2025 | Q15 | publication bias | No | Publication bias assessment was not reported. |
| R03 | Aleid 2025 | Q16 | conflict of interest | Yes | The review reported no conflicts of interest and disclosed financial support. |
| R04 | Chen 2024 | Q1 | PICO components | Yes | PICOS criteria specified PTBICD population, rTMS plus cognitive training, comparators, cognitive outcomes and RCT design. |
| R04 | Chen 2024 | Q2 | protocol registered before review | Yes | The meta-analysis was registered in PROSPERO (CRD42024520596). |
| R04 | Chen 2024 | Q3 | explanation of study designs | Yes | The review explicitly included RCTs and excluded non-RCTs, cross-over RCTs, reviews and conference abstracts. |
| R04 | Chen 2024 | Q4 | comprehensive search strategy | Yes | Eight English and Chinese databases were searched from inception to December 20, 2023, with keywords and full strategies reported in supplementary files. |
| R04 | Chen 2024 | Q5 | study selection duplicate | No | Duplicate independent study screening/selection was not explicitly reported. |
| R04 | Chen 2024 | Q6 | data extraction duplicate | Yes | Two reviewers independently extracted data, with disagreements resolved by discussion or arbitration. |
| R04 | Chen 2024 | Q7 | excluded studies list | No | Exclusion reasons were summarized but no list of excluded full-text studies with study-level reasons was provided. |
| R04 | Chen 2024 | Q8 | included studies description | Yes | Included RCT characteristics, interventions, outcomes, PEDro ratings and risk-of-bias results were described. |
| R04 | Chen 2024 | Q9 | risk of bias assessment | Yes | PEDro and Cochrane risk-of-bias assessment were performed independently. |
| R04 | Chen 2024 | Q10 | funding sources of primary studies | No | Funding sources of included primary studies were not reported. |
| R04 | Chen 2024 | Q11 | meta analysis methods | Yes | Meta-analytic models, heterogeneity thresholds, subgroup/sensitivity approaches and Stata analyses were reported. |
| R04 | Chen 2024 | Q12 | impact of RoB on results | Partial Yes | Risk of bias and GRADE certainty were assessed, but direct impact of RoB on pooled results was only partially addressed. |
| R04 | Chen 2024 | Q13 | RoB considered in interpretation | Yes | The review interpreted findings in light of study quality, GRADE certainty, sample size and bias concerns. |
| R04 | Chen 2024 | Q14 | heterogeneity explained | Yes | Heterogeneity was quantified and explored using sensitivity, Galbraith and subgroup analyses. |
| R04 | Chen 2024 | Q15 | publication bias | Yes | Funnel plot and Egger test were used for MoCA where enough RCTs were available. |
| R04 | Chen 2024 | Q16 | conflict of interest | Yes | Funding support and no competing interests were declared. |
| R05 | Nousia 2022 | Q1 | PICO components | Yes | Eligibility criteria specified TBI, NIBS/NIBS plus cognitive training, controlled RCTs and pre-post cognitive data. |
| R05 | Nousia 2022 | Q2 | protocol registered before review | No | The review explicitly stated that the protocol was not registered with an online database. |
| R05 | Nousia 2022 | Q3 | explanation of study designs | Yes | RCT design was required and other designs, protocols, abstracts and animal studies were excluded. |
| R05 | Nousia 2022 | Q4 | comprehensive search strategy | Partial Yes | PubMed and Google Scholar were searched with reported terms and reference/citation screening, but database coverage was limited and English-only restrictions were used. |
| R05 | Nousia 2022 | Q5 | study selection duplicate | Yes | Two independent reviewers conducted the literature search and review processes, with discrepancies resolved by a third author. |
| R05 | Nousia 2022 | Q6 | data extraction duplicate | Yes | Two independent reviewers performed data extraction independently. |
| R05 | Nousia 2022 | Q7 | excluded studies list | No | No list of excluded full-text studies with reasons was reported. |
| R05 | Nousia 2022 | Q8 | included studies description | Yes | Study characteristics, interventions, cognitive domains and risk-of-bias information were summarized. |
| R05 | Nousia 2022 | Q9 | risk of bias assessment | Yes | Cochrane RoB tool was used across standard bias domains. |
| R05 | Nousia 2022 | Q10 | funding sources of primary studies | No | Funding sources of included primary studies were not reported. |
| R05 | Nousia 2022 | Q11 | meta analysis methods | Not Applicable | No meta-analysis was conducted. |
| R05 | Nousia 2022 | Q12 | impact of RoB on results | Not Applicable | No meta-analysis was conducted. |
| R05 | Nousia 2022 | Q13 | RoB considered in interpretation | Yes | The discussion emphasized high risk of bias and methodological limitations when interpreting findings. |
| R05 | Nousia 2022 | Q14 | heterogeneity explained | Yes | Clinical and methodological heterogeneity in interventions, tests and outcomes was discussed. |
| R05 | Nousia 2022 | Q15 | publication bias | Not Applicable | No meta-analysis was conducted. |
| R05 | Nousia 2022 | Q16 | conflict of interest | Yes | The authors declared no conflicts of interest. |
| R06 | Hara 2021 | Q1 | PICO components | Yes | Eligibility criteria specified TBI patients, NIBS for cognitive rehabilitation, cognitive outcomes and RCT/PCT designs. |
| R06 | Hara 2021 | Q2 | protocol registered before review | Yes | A protocol was prospectively registered in PROSPERO (CRD42020183298). |
| R06 | Hara 2021 | Q3 | explanation of study designs | Yes | RCTs and prospective controlled trials were eligible; protocols, case reports and retrospective studies were excluded. |
| R06 | Hara 2021 | Q4 | comprehensive search strategy | Partial Yes | Six databases and an example PubMed/MEDLINE strategy were reported; language was restricted to English and a full excluded-search strategy for all databases was not provided in the main text. |
| R06 | Hara 2021 | Q5 | study selection duplicate | Yes | Two authors independently reviewed studies for eligibility, with a third author resolving disagreements. |
| R06 | Hara 2021 | Q6 | data extraction duplicate | Yes | Two authors independently extracted data using a standard form, with discrepancies checked and adjudicated. |
| R06 | Hara 2021 | Q7 | excluded studies list | No | A flow diagram and exclusion categories were reported, but no study-level excluded full-text list was provided. |
| R06 | Hara 2021 | Q8 | included studies description | Yes | Tables described study characteristics, treatments, outcomes, results and adverse events. |
| R06 | Hara 2021 | Q9 | risk of bias assessment | Yes | Methodological quality was assessed using the PEDro scoring system and level of evidence. |
| R06 | Hara 2021 | Q10 | funding sources of primary studies | No | Funding sources of included primary studies were not reported. |
| R06 | Hara 2021 | Q11 | meta analysis methods | Not Applicable | No meta-analysis was conducted. |
| R06 | Hara 2021 | Q12 | impact of RoB on results | Not Applicable | No meta-analysis was conducted. |
| R06 | Hara 2021 | Q13 | RoB considered in interpretation | Yes | Evidence limitations and methodological quality were considered in the interpretation. |
| R06 | Hara 2021 | Q14 | heterogeneity explained | Yes | The review discussed heterogeneity in stimulation parameters, targets and neuropsychological tests. |
| R06 | Hara 2021 | Q15 | publication bias | Not Applicable | No meta-analysis was conducted. |
| R06 | Hara 2021 | Q16 | conflict of interest | Partial Yes | Funding was disclosed, but a separate competing-interest statement was not clearly identified in the extracted text. |
| R07 | Tsai 2021 | Q1 | PICO components | Yes | Eligibility criteria specified TBI population, rTMS intervention, sham/control comparison, depression/cognition/PCS outcomes and RCT design. |
| R07 | Tsai 2021 | Q2 | protocol registered before review | Yes | The meta-analysis was registered in PROSPERO (CRD42020200348). |
| R07 | Tsai 2021 | Q3 | explanation of study designs | Yes | Only RCTs were eligible, with explicit inclusion/exclusion criteria. |
| R07 | Tsai 2021 | Q4 | comprehensive search strategy | Partial Yes | Five databases were searched, but the reported search string was narrow and full database-specific strategies were not shown. |
| R07 | Tsai 2021 | Q5 | study selection duplicate | Yes | Two authors independently screened titles and abstracts, with third-reviewer consensus for discrepancies. |
| R07 | Tsai 2021 | Q6 | data extraction duplicate | No | Data extraction was described by reviewers, but independent duplicate or checked extraction was not clearly reported. |
| R07 | Tsai 2021 | Q7 | excluded studies list | No | No excluded full-text study list with reasons was provided. |
| R07 | Tsai 2021 | Q8 | included studies description | Yes | Tables summarized population, intervention, outcomes and rTMS protocols. |
| R07 | Tsai 2021 | Q9 | risk of bias assessment | Yes | RoB 2.0 was used and study quality concerns were summarized. |
| R07 | Tsai 2021 | Q10 | funding sources of primary studies | No | Funding sources of primary studies were not reported. |
| R07 | Tsai 2021 | Q11 | meta analysis methods | Yes | Random-effects meta-analysis, SMDs and I2 heterogeneity were described. |
| R07 | Tsai 2021 | Q12 | impact of RoB on results | Partial Yes | Risk-of-bias findings were described, but their effect on pooled estimates was not formally tested. |
| R07 | Tsai 2021 | Q13 | RoB considered in interpretation | Partial Yes | Study limitations and quality issues were discussed, but RoB was only partly integrated into interpretation. |
| R07 | Tsai 2021 | Q14 | heterogeneity explained | Yes | The review discussed heterogeneity in protocols, target sites, duration and outcome measures. |
| R07 | Tsai 2021 | Q15 | publication bias | No | Publication bias or small-study effects were not reported. |
| R07 | Tsai 2021 | Q16 | conflict of interest | Yes | The authors declared no competing interests. |
| R08 | Kawata 2024 | Q1 | PICO components | Partial Yes | The population, RCT design and broad intervention/outcome categories were described, but the review was broad rather than cognition/NIBS-specific. |
| R08 | Kawata 2024 | Q2 | protocol registered before review | No | No protocol registration or prior protocol was reported. |
| R08 | Kawata 2024 | Q3 | explanation of study designs | Yes | The review explicitly included RCTs and excluded cohort, case, animal, editorial and conference-abstract reports. |
| R08 | Kawata 2024 | Q4 | comprehensive search strategy | Partial Yes | Three databases, time frame, keywords and cited-paper screening were reported, but English-only restriction and limited database coverage reduce comprehensiveness. |
| R08 | Kawata 2024 | Q5 | study selection duplicate | Yes | Two independent reviewers performed identification, screening, eligibility and inclusion, with disagreements settled by a senior author. |
| R08 | Kawata 2024 | Q6 | data extraction duplicate | Yes | Two authors independently performed data extraction and quality assessment. |
| R08 | Kawata 2024 | Q7 | excluded studies list | No | Exclusion categories were reported, but no study-level excluded full-text list with reasons was provided. |
| R08 | Kawata 2024 | Q8 | included studies description | Yes | Study tables described RCT populations, interventions, time since injury and outcomes across domains. |
| R08 | Kawata 2024 | Q9 | risk of bias assessment | Yes | Cochrane risk-of-bias tool was used. |
| R08 | Kawata 2024 | Q10 | funding sources of primary studies | No | Funding sources of included primary studies were not reported. |
| R08 | Kawata 2024 | Q11 | meta analysis methods | Not Applicable | No meta-analysis was conducted. |
| R08 | Kawata 2024 | Q12 | impact of RoB on results | Not Applicable | No meta-analysis was conducted. |
| R08 | Kawata 2024 | Q13 | RoB considered in interpretation | Yes | Risk of bias, small samples and limitations were considered in the discussion. |
| R08 | Kawata 2024 | Q14 | heterogeneity explained | Yes | The review discussed heterogeneity across intervention types and trial populations. |
| R08 | Kawata 2024 | Q15 | publication bias | Not Applicable | No meta-analysis was conducted; publication bias was discussed narratively. |
| R08 | Kawata 2024 | Q16 | conflict of interest | Yes | Potential conflicts for one author were disclosed and remaining authors declared no relevant conflicts. |
| R09 | Zaninotto 2019 | Q1 | PICO components | Partial Yes | The review question specified adult TBI patients receiving tDCS and clinical/surrogate outcomes, but comparator and outcome domains were broad. |
| R09 | Zaninotto 2019 | Q2 | protocol registered before review | No | No protocol registration or prior protocol was reported. |
| R09 | Zaninotto 2019 | Q3 | explanation of study designs | Partial Yes | Experimental clinical trials, open-label studies and case reports were eligible; rationale for mixing designs was limited. |
| R09 | Zaninotto 2019 | Q4 | comprehensive search strategy | Partial Yes | MEDLINE/PubMed and Web of Science search strings and filters were reported, but only two databases and English-language filtering were used. |
| R09 | Zaninotto 2019 | Q5 | study selection duplicate | Yes | Two independent researchers reviewed titles and abstracts. |
| R09 | Zaninotto 2019 | Q6 | data extraction duplicate | No | Duplicate data extraction was not clearly reported. |
| R09 | Zaninotto 2019 | Q7 | excluded studies list | No | Flow counts were reported, but no list of excluded full-text studies with reasons was provided. |
| R09 | Zaninotto 2019 | Q8 | included studies description | Yes | A table described design, sample size, montage, stimulation parameters, outcomes and results. |
| R09 | Zaninotto 2019 | Q9 | risk of bias assessment | Partial Yes | Jadad score and funding-source bias were considered, but case reports/open-label designs were included and the RoB approach was limited for non-RCTs. |
| R09 | Zaninotto 2019 | Q10 | funding sources of primary studies | Partial Yes | The authors stated they evaluated funding sources for bias, but study-level funding details were not fully tabulated. |
| R09 | Zaninotto 2019 | Q11 | meta analysis methods | Not Applicable | No meta-analysis was conducted. |
| R09 | Zaninotto 2019 | Q12 | impact of RoB on results | Not Applicable | No meta-analysis was conducted. |
| R09 | Zaninotto 2019 | Q13 | RoB considered in interpretation | Partial Yes | Methodological variability and Jadad scores were discussed, but RoB was only partly integrated into conclusions. |
| R09 | Zaninotto 2019 | Q14 | heterogeneity explained | Yes | The review discussed heterogeneity in outcomes, designs and tDCS parameters. |
| R09 | Zaninotto 2019 | Q15 | publication bias | Not Applicable | No meta-analysis was conducted. |
| R09 | Zaninotto 2019 | Q16 | conflict of interest | Yes | The authors reported no conflicts of interest. |
| R10 | Oktaviani 2026 | Q1 | PICO components | Yes | Eligibility criteria specified adult TBI patients, rTMS intervention, sham/control groups, neuropsychiatric/pain/cognitive outcomes and RCT design. |
| R10 | Oktaviani 2026 | Q2 | protocol registered before review | No | No protocol registration or prior protocol was reported. |
| R10 | Oktaviani 2026 | Q3 | explanation of study designs | Yes | The review explicitly included RCTs and excluded non-randomized studies, reviews, editorials, animal and in vitro studies. |
| R10 | Oktaviani 2026 | Q4 | comprehensive search strategy | Partial Yes | Three databases, detailed search terms, reference-list screening and EndNote deduplication were reported; database coverage and English-only restriction limited comprehensiveness. |
| R10 | Oktaviani 2026 | Q5 | study selection duplicate | Yes | Three independent reviewers screened titles and abstracts, with disagreements resolved by discussion or consultation. |
| R10 | Oktaviani 2026 | Q6 | data extraction duplicate | No | Data items were described, but duplicate independent data extraction was not clearly reported. |
| R10 | Oktaviani 2026 | Q7 | excluded studies list | No | Flow counts and exclusion categories were reported, but no study-level excluded full-text list was provided. |
| R10 | Oktaviani 2026 | Q8 | included studies description | Yes | A table described study design, sample size, outcomes, instruments, results and follow-up. |
| R10 | Oktaviani 2026 | Q9 | risk of bias assessment | Yes | RoB 2.0 was independently applied by two reviewers. |
| R10 | Oktaviani 2026 | Q10 | funding sources of primary studies | No | Funding sources of included primary studies were not reported. |
| R10 | Oktaviani 2026 | Q11 | meta analysis methods | Yes | Random-effects meta-analysis, SMDs, I2, sensitivity analyses and TSA for significant pooled outcomes were described. |
| R10 | Oktaviani 2026 | Q12 | impact of RoB on results | Partial Yes | RoB was assessed and discussed, but its direct impact on pooled results was not fully examined. |
| R10 | Oktaviani 2026 | Q13 | RoB considered in interpretation | Yes | Interpretation considered RoB, heterogeneity, small samples and limited high-quality RCTs. |
| R10 | Oktaviani 2026 | Q14 | heterogeneity explained | Yes | Clinical/methodological heterogeneity in protocols, target regions and outcomes was discussed. |
| R10 | Oktaviani 2026 | Q15 | publication bias | Partial Yes | The review specified funnel/Egger methods if more than 10 studies were available and acknowledged that publication bias could not be thoroughly evaluated because of few studies. |
| R10 | Oktaviani 2026 | Q16 | conflict of interest | Yes | Financial support was nil and no conflicts of interest were declared. |
