# Supplementary material for: Non-invasive brain stimulation for cognitive impairment after traumatic brain injury: an umbrella review of systematic reviews
Source: Front Neurol. 2026 Aug 19;17:1931683. doi: 10.3389/fneur.2026.1931683 (PMC13533710; doi:10.3389/fneur.2026.1931683)
Supplement: Supplementary file 3 [file Table_3.DOCX]

**Supplementary Table S3. Overlap/citation matrix and corrected covered area assessment.**

Cells marked 1 indicate that the primary study was included in the corresponding review. The matrix includes 42 unique primary studies and 92 total review-level occurrences.

| **unique_primary_study_id** | **primary_first_author** | **primary_year** | **primary_title** | **R01** | **R02** | **R03** | **R04** | **R05** | **R06** | **R07** | **R08** | **R09** | **R10** | **number_of_reviews** |
| --- | --- | --- | --- | --- | --- | --- | --- | --- | --- | --- | --- | --- | --- | --- |
| UPS001 | Angelakis | 2014 | Transcranial Direct Current Stimulation Effects in Disorders of Consciousness | 0 | 0 | 0 | 0 | 0 | 0 | 0 | 0 | 1 | 0 | 1 |
| UPS002 | Bai | 2017 | TDCS modulates cortical excitability in patients with disorders of consciousness | 0 | 0 | 0 | 0 | 0 | 0 | 0 | 0 | 1 | 0 | 1 |
| UPS003 | Choi | 2018 | Effect of high-frequency repetitive transcranial magnetic stimulation on chronic central pain after mild traumatic brain injury: A pilot study | 0 | 0 | 1 | 0 | 0 | 0 | 0 | 1 | 0 | 1 | 3 |
| UPS004 | Estraneo | 2017 | Repeated transcranial direct current stimulation in prolonged disorders of consciousness: A double-blind cross-over study | 0 | 0 | 0 | 0 | 0 | 0 | 0 | 0 | 1 | 0 | 1 |
| UPS005 | Franke | 2022 | Randomized trial of rTMS in traumatic brain injury: Improved subjective neurobehavioral symptoms and increases in EEG delta activity | 0 | 0 | 1 | 0 | 0 | 0 | 0 | 0 | 0 | 1 | 2 |
| UPS006 | Guan | 2016 | Effect of low-frequency repetitive transcranial magnetic stimulation at different frequencies on cognitive function in patients with craniocerebral injury with a history of seizures | 0 | 0 | 0 | 1 | 0 | 0 | 0 | 0 | 0 | 0 | 1 |
| UPS007 | Hong/Fan | 2019 | Efficacy of low-frequency repetitive transcranial magnetic stimulation on cognitive function and activities of daily living after traumatic brain injury | 0 | 0 | 0 | 1 | 0 | 0 | 0 | 0 | 0 | 0 | 1 |
| UPS008 | Hoy | 2019 | A pilot investigation of repetitive transcranial magnetic stimulation for post-traumatic brain injury depression: Safety, tolerability, and efficacy | 1 | 1 | 1 | 0 | 1 | 0 | 1 | 1 | 0 | 1 | 7 |
| UPS009 | Huang | 2009 | Effect of low-frequency repetitive transcranial magnetic stimulation on cognitive function in patients with traumatic brain injury | 0 | 0 | 0 | 1 | 0 | 0 | 0 | 0 | 0 | 0 | 1 |
| UPS010 | Kang | 2012 | Transcranial direct current stimulation of the left prefrontal cortex improves attention in patients with traumatic brain injury: a pilot study | 0 | 0 | 0 | 0 | 1 | 0 | 0 | 1 | 1 | 0 | 3 |
| UPS011 | Koski | 2015 | Noninvasive brain stimulation for persistent postconcussion symptoms in mild traumatic brain injury | 0 | 1 | 1 | 0 | 0 | 0 | 0 | 0 | 0 | 0 | 2 |
| UPS012 | Lee | 2018 | Effect of low frequency repetitive transcranial magnetic stimulation on depression and cognition of patients with traumatic brain injury: A randomized controlled trial | 1 | 1 | 1 | 1 | 1 | 1 | 1 | 1 | 0 | 1 | 9 |
| UPS013 | Lesniak | 2014 | Effects of repeated anodal tDCS coupled with cognitive training for patients with severe traumatic brain injury: A pilot randomized controlled trial | 1 | 1 | 0 | 0 | 1 | 1 | 0 | 1 | 1 | 0 | 6 |
| UPS014 | Leung | 2016 | Repetitive Transcranial Magnetic Stimulation in Managing Mild Traumatic Brain Injury-Related Headaches | 1 | 0 | 0 | 0 | 0 | 0 | 0 | 0 | 0 | 1 | 2 |
| UPS015 | Leung | 2018 | Left dorsolateral prefrontal cortex rTMS in alleviating MTBI related headaches and depressive symptoms | 0 | 0 | 0 | 0 | 0 | 0 | 1 | 0 | 0 | 0 | 1 |
| UPS016 | Liao | 2017 | Efficacy of repetitive transcranial magnetic stimulation combined with ocular therapy for cognitive dysfunction in patients with traumatic brain injury | 0 | 0 | 0 | 1 | 0 | 0 | 0 | 0 | 0 | 0 | 1 |
| UPS017 | Martens | 2018 | Randomized controlled trial of home-based 4-week tDCS in chronic minimally conscious state | 0 | 0 | 0 | 0 | 0 | 0 | 0 | 0 | 1 | 0 | 1 |
| UPS018 | Middleton | 2014 | Using clinical and robotic assessment tools to examine the feasibility of pairing tDCS with upper extremity physical therapy in patients with stroke and TBI: A consideration-of-concept pilot study | 0 | 0 | 0 | 0 | 0 | 0 | 0 | 0 | 1 | 0 | 1 |
| UPS019 | Motes | 2020 | High-Definition Transcranial Direct Current Stimulation to Improve Verbal Retrieval Deficits in Chronic Traumatic Brain Injury | 1 | 0 | 0 | 0 | 0 | 0 | 0 | 0 | 0 | 0 | 1 |
| UPS020 | Moussavi | 2019 | A pilot randomised double-blind study of the tolerability and efficacy of repetitive transcranial magnetic stimulation on persistent post-concussion syndrome | 0 | 0 | 0 | 0 | 1 | 0 | 1 | 1 | 0 | 1 | 4 |
| UPS021 | Naro | 2015 | Can transcranial direct current stimulation be useful in differentiating unresponsive wakefulness syndrome from minimally conscious state patients? | 0 | 0 | 0 | 0 | 0 | 0 | 0 | 0 | 1 | 0 | 1 |
| UPS022 | Neville | 2019 | Repetitive TMS does not improve cognition in patients with TBI: a randomized double-blind trial | 1 | 1 | 0 | 1 | 1 | 1 | 0 | 1 | 0 | 1 | 7 |
| UPS023 | O'Neil-Pirozzi | 2017 | Immediate memory and electrophysiologic effects of prefrontal cortex transcranial direct current stimulation on neurotypical individuals and individuals with chronic traumatic brain injury: a pilot study | 0 | 0 | 0 | 0 | 0 | 0 | 0 | 0 | 1 | 0 | 1 |
| UPS024 | Quinn | 2020 | NR | 0 | 1 | 0 | 0 | 0 | 0 | 0 | 0 | 0 | 0 | 1 |
| UPS025 | Rao | 2019 | Low-frequency right repetitive transcranial magnetic stimulation for the treatment of depression after traumatic brain injury: A randomized sham-controlled pilot study | 1 | 0 | 0 | 0 | 1 | 0 | 1 | 0 | 0 | 1 | 4 |
| UPS026 | Rodrigues | 2020 | The effects of repetitive transcranial magnetic stimulation on anxiety in patients with moderate to severe traumatic brain injury: A post-hoc analysis of a randomized clinical trial | 0 | 0 | 0 | 0 | 0 | 0 | 0 | 0 | 0 | 1 | 1 |
| UPS027 | Rushby | 2020 | tDCS effects on task-related activation and working memory performance in traumatic brain injury: A within-group randomized controlled trial | 0 | 0 | 0 | 0 | 1 | 0 | 0 | 0 | 0 | 0 | 1 |
| UPS028 | Rutherford | 2017 | NR | 0 | 1 | 0 | 0 | 0 | 0 | 0 | 0 | 0 | 0 | 1 |
| UPS029 | Sacco | 2016 | Concomitant use of transcranial direct current stimulation and computer-assisted training for the rehabilitation of attention in traumatic brain injured patients: Behavioral and neuroimaging results | 0 | 1 | 0 | 0 | 1 | 1 | 0 | 0 | 1 | 0 | 4 |
| UPS030 | Shen | 2023 | Effect of high-frequency repetitive transcranial magnetic stimulation over M1 for consciousness recovery after traumatic brain injury | 0 | 0 | 1 | 0 | 0 | 0 | 0 | 0 | 0 | 0 | 1 |
| UPS031 | Siddiqi | 2019 | Repetitive transcranial magnetic stimulation with resting-state network targeting for treatment-resistant depression in traumatic brain injury: a randomized, controlled, double-blinded pilot study | 1 | 0 | 0 | 0 | 0 | 0 | 1 | 1 | 0 | 1 | 4 |
| UPS032 | Stilling | 2020 | Treatment of persistent post-traumatic headache and post-concussion symptoms using repetitive transcranial magnetic stimulation: A pilot, double-blind randomized controlled trial | 0 | 0 | 1 | 0 | 0 | 0 | 1 | 0 | 0 | 1 | 3 |
| UPS033 | Sun | 2022 | Efficacy of low-frequency repetitive transcranial magnetic stimulation on cognitive function and activities of daily living after traumatic brain injury | 0 | 0 | 0 | 1 | 0 | 0 | 0 | 0 | 0 | 0 | 1 |
| UPS034 | Thibaut | 2014 | tDCS in patients with disorders of consciousness | 0 | 0 | 0 | 0 | 0 | 0 | 0 | 0 | 1 | 0 | 1 |
| UPS035 | Thibaut | 2017 | Controlled clinical trial of repeated prefrontal tDCS in patients with chronic minimally conscious state | 0 | 0 | 0 | 0 | 0 | 0 | 0 | 0 | 1 | 0 | 1 |
| UPS036 | Ulam | 2015 | Cumulative effects of transcranial direct current stimulation on EEG oscillations and attention/working memory during subacute neurorehabilitation of traumatic brain injury | 1 | 1 | 0 | 0 | 1 | 1 | 0 | 0 | 1 | 0 | 5 |
| UPS037 | Wang | 2017 | Effect of repetitive transcranial magnetic stimulation on memory and quality of life in patients with traumatic brain injury | 0 | 0 | 0 | 1 | 0 | 0 | 0 | 0 | 0 | 0 | 1 |
| UPS038 | Wilke | 2017 | No Effect of Anodal Transcranial Direct Current Stimulation on Gamma-Aminobutyric Acid Levels in Patients with Recurrent Mild Traumatic Brain Injury | 0 | 0 | 0 | 0 | 0 | 0 | 0 | 0 | 1 | 0 | 1 |
| UPS039 | Zhai | 2023 | Effect of low-frequency repetitive transcranial magnetic stimulation on cognitive function and P300 potential after traumatic brain injury | 0 | 0 | 0 | 1 | 0 | 0 | 0 | 0 | 0 | 0 | 1 |
| UPS040 | Zhang | 2021 | Repetitive transcranial magnetic stimulation combined with rehabilitation training improves cognitive function in patients with craniocerebral injury | 0 | 0 | 0 | 1 | 0 | 0 | 0 | 0 | 0 | 0 | 1 |
| UPS041 | Zhao | 2020 | Effects of high- and low-frequency repetitive transcranial magnetic stimulation on cognitive function after traumatic brain injury | 0 | 0 | 0 | 1 | 0 | 0 | 0 | 0 | 0 | 0 | 1 |
| UPS042 | Zhou | 2019 | Effect of high-frequency repetitive transcranial magnetic stimulation combined with cognitive training in patients with cognitive impairment after traumatic head injury | 0 | 0 | 0 | 1 | 0 | 0 | 0 | 0 | 0 | 0 | 1 |
