# Supplementary material for: Non-invasive brain stimulation for cognitive impairment after traumatic brain injury: an umbrella review of systematic reviews
Source: Front Neurol. 2026 Aug 19;17:1931683. doi: 10.3389/fneur.2026.1931683 (PMC13533710; doi:10.3389/fneur.2026.1931683)
Supplement: Supplementary file 4 [file Table_4.DOCX]

**Supplementary Table S4. Unique and repeated primary studies.**

Fitzgerald 2009 was removed from the main overlap analysis and retained for manual checking because the TBI population was unclear. The table below contains the 42 unique primary studies in the final main analysis.

| **unique_primary_study_id** | **primary_first_author** | **primary_year** | **primary_title** | **DOI** | **PMID** | **sample_size** |
| --- | --- | --- | --- | --- | --- | --- |
| UPS001 | Angelakis | 2014 | Transcranial Direct Current Stimulation Effects in Disorders of Consciousness | NR | NR | 10 total; 5 TBI |
| UPS002 | Bai | 2017 | TDCS modulates cortical excitability in patients with disorders of consciousness | NR | NR | 16 total; 4 VS/MCS due to TBI |
| UPS003 | Choi | 2018 | Effect of high-frequency repetitive transcranial magnetic stimulation on chronic central pain after mild traumatic brain injury: A pilot study | 10.2340/16501977-2321 | NR | 12 |
| UPS004 | Estraneo | 2017 | Repeated transcranial direct current stimulation in prolonged disorders of consciousness: A double-blind cross-over study | NR | NR | 23 total; 1 VS due to TBI |
| UPS005 | Franke | 2022 | Randomized trial of rTMS in traumatic brain injury: Improved subjective neurobehavioral symptoms and increases in EEG delta activity | NR | NR | NR |
| UPS006 | Guan | 2016 | Effect of low-frequency repetitive transcranial magnetic stimulation at different frequencies on cognitive function in patients with craniocerebral injury with a history of seizures | 10.3760/cma.j.issn.0254-1424.2016.05.007 | NR | 60 |
| UPS007 | Hong/Fan | 2019 | Efficacy of low-frequency repetitive transcranial magnetic stimulation on cognitive function and activities of daily living after traumatic brain injury | NR | NR | 51 |
| UPS008 | Hoy | 2019 | A pilot investigation of repetitive transcranial magnetic stimulation for post-traumatic brain injury depression: Safety, tolerability, and efficacy | 10.1089/neu.2018.6097 | NR | 21 |
| UPS009 | Huang | 2009 | Effect of low-frequency repetitive transcranial magnetic stimulation on cognitive function in patients with traumatic brain injury | NR | NR | 60 |
| UPS010 | Kang | 2012 | Transcranial direct current stimulation of the left prefrontal cortex improves attention in patients with traumatic brain injury: a pilot study | 10.2340/16501977-0947 | NR | 9 |
| UPS011 | Koski | 2015 | Noninvasive brain stimulation for persistent postconcussion symptoms in mild traumatic brain injury | 10.1089/neu.2014.3449 | NR | 15 |
| UPS012 | Lee | 2018 | Effect of low frequency repetitive transcranial magnetic stimulation on depression and cognition of patients with traumatic brain injury: A randomized controlled trial | 10.12659/msm.911385 | NR | 13 |
| UPS013 | Lesniak | 2014 | Effects of repeated anodal tDCS coupled with cognitive training for patients with severe traumatic brain injury: A pilot randomized controlled trial | 10.1097/htr.0b013e318292a4c2 | NR | 23 |
| UPS014 | Leung | 2016 | Repetitive Transcranial Magnetic Stimulation in Managing Mild Traumatic Brain Injury-Related Headaches | NR | NR | 24 |
| UPS015 | Leung | 2018 | Left dorsolateral prefrontal cortex rTMS in alleviating MTBI related headaches and depressive symptoms | 10.1111/ner.12615 | NR | 29 |
| UPS016 | Liao | 2017 | Efficacy of repetitive transcranial magnetic stimulation combined with ocular therapy for cognitive dysfunction in patients with traumatic brain injury | NR | NR | 60 |
| UPS017 | Martens | 2018 | Randomized controlled trial of home-based 4-week tDCS in chronic minimally conscious state | NR | NR | 27 total; 12 DOC due to TBI |
| UPS018 | Middleton | 2014 | Using clinical and robotic assessment tools to examine the feasibility of pairing tDCS with upper extremity physical therapy in patients with stroke and TBI: A consideration-of-concept pilot study | NR | NR | 5 total; 2 TBI |
| UPS019 | Motes | 2020 | High-Definition Transcranial Direct Current Stimulation to Improve Verbal Retrieval Deficits in Chronic Traumatic Brain Injury | NR | NR | 15 |
| UPS020 | Moussavi | 2019 | A pilot randomised double-blind study of the tolerability and efficacy of repetitive transcranial magnetic stimulation on persistent post-concussion syndrome | 10.1038/s41598-019-41923-6 | NR | 8 severe PCS subset in the Tsai review table; original trial N=18 |
| UPS021 | Naro | 2015 | Can transcranial direct current stimulation be useful in differentiating unresponsive wakefulness syndrome from minimally conscious state patients? | NR | NR | 45 total; 25 DOC due to TBI or anoxia |
| UPS022 | Neville | 2019 | Repetitive TMS does not improve cognition in patients with TBI: a randomized double-blind trial | 10.1212/wnl.0000000000007748 | NR | 30 |
| UPS023 | O'Neil-Pirozzi | 2017 | Immediate memory and electrophysiologic effects of prefrontal cortex transcranial direct current stimulation on neurotypical individuals and individuals with chronic traumatic brain injury: a pilot study | NR | NR | 8 total; 4 chronic severe TBI |
| UPS024 | Quinn | 2020 | NR | NR | NR | 24 |
| UPS025 | Rao | 2019 | Low-frequency right repetitive transcranial magnetic stimulation for the treatment of depression after traumatic brain injury: A randomized sham-controlled pilot study | 10.1176/appi.neuropsych.17110338 | NR | 30 |
| UPS026 | Rodrigues | 2020 | The effects of repetitive transcranial magnetic stimulation on anxiety in patients with moderate to severe traumatic brain injury: A post-hoc analysis of a randomized clinical trial | NR | NR | 30 |
| UPS027 | Rushby | 2020 | tDCS effects on task-related activation and working memory performance in traumatic brain injury: A within-group randomized controlled trial | NR | NR | 30 |
| UPS028 | Rutherford | 2017 | NR | NR | NR | 17 |
| UPS029 | Sacco | 2016 | Concomitant use of transcranial direct current stimulation and computer-assisted training for the rehabilitation of attention in traumatic brain injured patients: Behavioral and neuroimaging results | NR | NR | 32 |
| UPS030 | Shen | 2023 | Effect of high-frequency repetitive transcranial magnetic stimulation over M1 for consciousness recovery after traumatic brain injury | NR | NR | NR |
| UPS031 | Siddiqi | 2019 | Repetitive transcranial magnetic stimulation with resting-state network targeting for treatment-resistant depression in traumatic brain injury: a randomized, controlled, double-blinded pilot study | 10.1089/neu.2018.5889 | NR | 15 |
| UPS032 | Stilling | 2020 | Treatment of persistent post-traumatic headache and post-concussion symptoms using repetitive transcranial magnetic stimulation: A pilot, double-blind randomized controlled trial | 10.1089/neu.2019.6692 | NR | 20 |
| UPS033 | Sun | 2022 | Efficacy of low-frequency repetitive transcranial magnetic stimulation on cognitive function and activities of daily living after traumatic brain injury | 10.15971/j.cnki.cmdi.2022.04.015 | NR | 82 |
| UPS034 | Thibaut | 2014 | tDCS in patients with disorders of consciousness | NR | NR | 55 total; 32 DOC due to TBI |
| UPS035 | Thibaut | 2017 | Controlled clinical trial of repeated prefrontal tDCS in patients with chronic minimally conscious state | NR | NR | 21 total; 11 of 16 DOC due to TBI |
| UPS036 | Ulam | 2015 | Cumulative effects of transcranial direct current stimulation on EEG oscillations and attention/working memory during subacute neurorehabilitation of traumatic brain injury | NR | NR | 26 |
| UPS037 | Wang | 2017 | Effect of repetitive transcranial magnetic stimulation on memory and quality of life in patients with traumatic brain injury | NR | NR | 60 |
| UPS038 | Wilke | 2017 | No Effect of Anodal Transcranial Direct Current Stimulation on Gamma-Aminobutyric Acid Levels in Patients with Recurrent Mild Traumatic Brain Injury | NR | NR | 39 total; 17 chronic mild TBI |
| UPS039 | Zhai | 2023 | Effect of low-frequency repetitive transcranial magnetic stimulation on cognitive function and P300 potential after traumatic brain injury | 10.20047/j.issn1673-7210.2023.26.20 | NR | 50 |
| UPS040 | Zhang | 2021 | Repetitive transcranial magnetic stimulation combined with rehabilitation training improves cognitive function in patients with craniocerebral injury | NR | NR | 80 |
| UPS041 | Zhao | 2020 | Effects of high- and low-frequency repetitive transcranial magnetic stimulation on cognitive function after traumatic brain injury | NR | NR | 60 |
| UPS042 | Zhou | 2019 | Effect of high-frequency repetitive transcranial magnetic stimulation combined with cognitive training in patients with cognitive impairment after traumatic head injury | NR | NR | 64 |

| **unique_primary_study_id** | **TBI_population** | **intervention** | **comparator** | **cognitive_outcomes** | **included_in_reviews** | **number_of_reviews_including_this_study** | **duplicate_resolution_method** | **notes** |
| --- | --- | --- | --- | --- | --- | --- | --- | --- |
| UPS001 | DOC due to TBI subset | open-label anodal tDCS over C3 or F3 with dose escalation | sham week followed by active tDCS dose escalation | CRS-R consciousness outcome; not cognition-specific | R09 | 1 | single record; no duplicate detected | mixed DOC/consciousness outcome; TBI subset only |
| UPS002 | VS/MCS due to TBI subset | anodal tDCS over F3, 2 mA for 20 min, cross-over | sham tDCS | TMS MEP and EEG; consciousness/neurophysiology outcome | R09 | 1 | single record; no duplicate detected | DOC/neurophysiology outcome; TBI subset only |
| UPS003 | adults with mild TBI and chronic central pain | 10 Hz rTMS over affected precentral gyrus, 10 sessions | sham rTMS | no cognitive outcome; central pain and clinical outcomes | R03; R08; R10 | 3 | probable duplicate: same first author/year and highly similar title | RCT; cognition not reported; possible duplicate across R06-R10 reviews; possible duplicate with R01-R05 (R03) \| pilot RCT; TBI+rTMS but cognition not reported; possible duplicate across R06-R10 reviews; possible duplicate with R01-R05 (R03); merged 3 review-level rows into one unique primary study |
| UPS004 | prolonged DOC; 1 TBI VS patient | anodal tDCS over F3, 2 mA for 20 min, cross-over | sham tDCS | CRS-R and EEG; consciousness outcome | R09 | 1 | single record; no duplicate detected | very small TBI subset; DOC/consciousness outcome |
| UPS005 | TBI patients | rTMS | sham/control | cognitive/neurobehavioral symptoms | R03; R10 | 2 | probable duplicate: same first author/year and highly similar title | RCT; neurobehavioral symptoms, cognition not separately specified; possible duplicate with R01-R05 (R03); merged 2 review-level rows into one unique primary study |
| UPS006 | craniocerebral injury patients with seizure history/cognitive dysfunction | rTMS + cognitive training | sham rTMS + cognitive training | MoCA; RBMT; adverse reactions | R04 | 1 | single record; no duplicate detected | NR |
| UPS007 | PTBICD patients | rTMS + cognitive training | cognitive training | MMSE; MBI; adverse reactions | R04 | 1 | single record; no duplicate detected | NR |
| UPS008 | TBI patients with post-traumatic brain injury depression | bilateral DLPFC rTMS | sham rTMS | attention; executive function; memory | R01; R02; R03; R05; R07; R08; R10 | 7 | DOI exact match; probable title/author matching for records without DOI where applicable | RCT; cognition secondary; possible duplicate across R06-R10 reviews; possible duplicate with R01-R05 (R01, R02, R03, R05) \| pilot RCT; cognitive outcomes reported as working memory/executive function; possible duplicate across R06-R10 reviews; possible duplicate with R01-R05 (R01, R02, R03, R05) \| pilot RCT; cognitive outcomes reported; possible duplicate across R06-R10 reviews; possible duplicate with R01-R05 (R01, R02, R03, R05) \| possible duplicate across reviews; merged 7 review-level rows into one unique primary study |
| UPS009 | TBI cognitive disorder patients | rTMS + cognitive training | sham rTMS + cognitive training | MoCA; MBI | R04 | 1 | probable duplicate: same first author/year and highly similar title | possible duplicate across reviews \| unclear duplicate with PS021; table lists separate Huang et al. [24] and Huang et al. [37] entries; possible duplicate across reviews; merged 2 review-level rows into one unique primary study |
| UPS010 | adults with TBI | 2 mA tDCS over left DLPFC in cross-over design | sham tDCS | attention, fatigue, task difficulty, sleep quality ratings | R05; R08; R09 | 3 | probable duplicate: same first author/year and highly similar title | cross-over clinical study; no significant post-intervention attention difference in Kawata table; possible duplicate across R06-R10 reviews \| tDCS/TBI clinical study; cognitive attention outcome; possible duplicate across R06-R10 reviews; merged 3 review-level rows into one unique primary study |
| UPS011 | mild TBI with persistent postconcussion symptoms | left DLPFC rTMS | NR | general cognitive function; executive function; attention; verbal fluency; memory | R02; R03 | 2 | DOI exact match; probable title/author matching for records without DOI where applicable | possible duplicate across reviews; merged 2 review-level rows into one unique primary study |
| UPS012 | TBI patients | right DLPFC low-frequency rTMS | sham/control | executive function | R01; R02; R03; R04; R05; R06; R07; R08; R10 | 9 | DOI exact match; probable title/author matching for records without DOI where applicable | RCT; attention significantly improved compared with control; possible duplicate across R06-R10 reviews; possible duplicate with R01-R05 (R01, R02, R03, R04, R05) \| RCT; cognitive outcome reported; possible duplicate across R06-R10 reviews; possible duplicate with R01-R05 (R01, R02, R03, R04, R05) \| RCT; cognitive outcomes reported; possible duplicate across R06-R10 reviews; possible duplicate with R01-R05 (R01, R02, R03, R04, R05) \| possible duplicate across reviews \| single-blinded RCT; cognitive outcomes reported; possible duplicate across R06-R10 reviews; possible duplicate with R01-R05 (R01, R02, R03, R04, R05); merged 9 review-level rows into one unique primary study |
| UPS013 | severe TBI patients | left DLPFC anodal tDCS plus cognitive training | sham tDCS plus cognitive training | attention; memory | R01; R02; R05; R06; R08; R09 | 6 | DOI exact match; probable title/author matching for records without DOI where applicable | pilot RCT; combined tDCS+cognitive training, not tDCS alone; possible duplicate across R06-R10 reviews; possible duplicate with R01-R05 (R01, R02, R05) \| pilot RCT; combined tDCS+cognitive training; possible duplicate across R06-R10 reviews; possible duplicate with R01-R05 (R01, R02, R05) \| possible duplicate across reviews \| year reported as 2013 in Zaninotto table but article citation is 2014; verify year before final overlap merge; merged 6 review-level rows into one unique primary study |
| UPS014 | mild TBI-related headache patients | rTMS | sham/control | attention | R01; R10 | 2 | probable duplicate: same first author/year and highly similar title | RCT; cognition/neuropsychological function reported; possible duplicate with R01-R05 (R01); merged 2 review-level rows into one unique primary study |
| UPS015 | mild TBI with post-traumatic headache | left DLPFC rTMS | sham rTMS | cognition not directly reported in the Tsai review table | R07 | 1 | single record; no duplicate detected | RCT; cognition not shown in table |
| UPS016 | TBI cognitive dysfunction patients | rTMS + cognitive training | cognitive training plus eye acupuncture | LOTCA | R04 | 1 | single record; no duplicate detected | NR |
| UPS017 | chronic minimally conscious state; TBI subset | home-based anodal tDCS over F3, 2 mA for 20 min over 2-4 weeks | sham tDCS | adverse events, adherence, CRS-R; consciousness outcome | R09 | 1 | single record; no duplicate detected | DOC/consciousness outcome; TBI subset only |
| UPS018 | mixed stroke/TBI patients | ipsilesional anodal tDCS paired with upper extremity physical therapy | NR | motor outcomes: Fugl-Meyer, Purdue Pegboard, Box and Block, SIS-16; not cognition-specific | R09 | 1 | single record; no duplicate detected | mixed stroke/TBI sample; motor outcome; unclear relevance for cognition overlap |
| UPS019 | chronic TBI patients | HD-tDCS over preSMA/dACC | non-randomized control/pre-post comparator | attention; executive function; memory; language | R01 | 1 | single record; no duplicate detected | NR |
| UPS020 | TBI with persistent post-concussion symptoms; severe PCS subset used by Tsai | rTMS | sham rTMS | post-concussion symptoms; cognition not directly reported in the Tsai review table | R05; R07; R08; R10 | 4 | DOI exact match; probable title/author matching for records without DOI where applicable | RCT; cognition not separately reported; possible duplicate across R06-R10 reviews; possible duplicate with R01-R05 (R05) \| pilot double-blind RCT; Tsai included severe PCS subset; possible duplicate across R06-R10 reviews; possible duplicate with R01-R05 (R05) \| pilot double-blind RCT; cognition not separately reported; possible duplicate across R06-R10 reviews; possible duplicate with R01-R05 (R05); merged 4 review-level rows into one unique primary study |
| UPS021 | DOC due to TBI or anoxia subset | anodal tDCS over supraorbital region, 1 mA for 10 min | sham tDCS | CRS-R, MEP, ICF, ICI, SICI; consciousness/neurophysiology outcome | R09 | 1 | single record; no duplicate detected | mixed TBI/anoxia DOC sample; unclear TBI-only clinical effect |
| UPS022 | TBI patients | left DLPFC rTMS | sham rTMS | attention; executive function; memory; motor function | R01; R02; R04; R05; R06; R08; R10 | 7 | DOI exact match; probable title/author matching for records without DOI where applicable | RCT; cognitive outcome primary/reported; possible duplicate across R06-R10 reviews; possible duplicate with R01-R05 (R01, R02, R04, R05) \| RCT; no between-group cognitive benefit; possible duplicate across R06-R10 reviews; possible duplicate with R01-R05 (R01, R02, R04, R05) \| RCT; no significant between-group cognitive benefit reported; possible duplicate across R06-R10 reviews; possible duplicate with R01-R05 (R01, R02, R04, R05) \| possible duplicate across reviews; merged 7 review-level rows into one unique primary study |
| UPS023 | chronic severe TBI subset plus neurotypical controls | anodal, cathodal, or sham tDCS over F3, 2 mA for 20 min, cross-over | sham tDCS | working memory test, EEG alpha power, P300 amplitude/latency | R09 | 1 | single record; no duplicate detected | mixed TBI/control sample; cognitive electrophysiology outcome |
| UPS024 | mild and moderate TBI patients | left DLPFC anodal tDCS | sham tDCS | executive function; working memory; attention; verbal learning and memory; general cognition | R02 | 1 | single record; no duplicate detected | reference details not clearly located in PDF text |
| UPS025 | TBI with depression | rTMS | sham rTMS | TMT-A, TMT-B, BVMT, SCWT | R01; R05; R07; R10 | 4 | probable duplicate: same first author/year and highly similar title | RCT; sample size conflicts with other reviews, verify before pooled overlap; possible duplicate across R06-R10 reviews; possible duplicate with R01-R05 (R01, R05) \| pilot RCT; cognitive outcomes reported; possible duplicate across R06-R10 reviews; possible duplicate with R01-R05 (R01, R05) \| possible duplicate across reviews; merged 4 review-level rows into one unique primary study |
| UPS026 | moderate to severe TBI | rTMS | sham/control as reported | MoCA, WCST, Stroop, BVMT, HVLT; cognition reported | R10 | 1 | single record; no duplicate detected | post-hoc analysis of randomized clinical trial; include for overlap with caution |
| UPS027 | TBI patients | single-session tDCS over parietal cortex | sham tDCS | working memory; attention; processing speed | R05 | 1 | single record; no duplicate detected | NR |
| UPS028 | TBI patients; time since injury <6 months; severity NR | left DLPFC rTMS | sham | general cognitive function (MoCA) | R02 | 1 | single record; no duplicate detected | reference details not clearly located in PDF text |
| UPS029 | severe TBI patients | tDCS plus divided attention training | sham tDCS/control training | divided attention; visual-spatial abilities; semantic fluency; memory | R02; R05; R06; R09 | 4 | probable duplicate: same first author/year and highly similar title | RCT; mixed cognitive-domain effects; possible duplicate across R06-R10 reviews; possible duplicate with R01-R05 (R02, R05) \| possible duplicate across reviews \| tDCS+cognitive training; cognitive attention outcome; possible duplicate across R06-R10 reviews; possible duplicate with R01-R05 (R02, R05); merged 4 review-level rows into one unique primary study |
| UPS030 | TBI patients with consciousness impairment | high-frequency rTMS over M1 | sham/control | consciousness/cognitive function unclear | R03 | 1 | single record; no duplicate detected | NR |
| UPS031 | moderate TBI with treatment-resistant depression | bilateral DLPFC rTMS | sham rTMS | cognition not directly reported in the Tsai review table | R01; R07; R08; R10 | 4 | DOI exact match; probable title/author matching for records without DOI where applicable | pilot RCT; cognition not shown in the Tsai review table; possible duplicate across R06-R10 reviews; possible duplicate with R01-R05 (R01) \| pilot RCT; no group difference in cognitive battery reported; possible duplicate across R06-R10 reviews; possible duplicate with R01-R05 (R01) \| verified primary clinical study; randomized controlled double-blinded pilot study; primary outcome depression; cognitive outcomes secondary/exploratory if reported; merged 4 review-level rows into one unique primary study |
| UPS032 | mild TBI with post-traumatic headache and persistent post-concussion symptoms | rTMS | sham rTMS | post-concussion symptoms; cognition not directly reported in the Tsai review table | R03; R07; R10 | 3 | probable duplicate: same first author/year and highly similar title | RCT; cognition not reported in table; possible duplicate with R01-R05 (R03) \| RCT; cognition not separately reported; merged 3 review-level rows into one unique primary study |
| UPS033 | TBI cognitive disorder patients | rTMS + cognitive training | cognitive training | MoCA; MMSE; MBI; adverse reactions | R04 | 1 | single record; no duplicate detected | NR |
| UPS034 | DOC due to TBI subset | anodal tDCS over F3, 2 mA for 20 min, cross-over | sham tDCS | CRS-R, GOSe; consciousness outcome | R09 | 1 | single record; no duplicate detected | mixed DOC/consciousness outcome; TBI subset only |
| UPS035 | chronic minimally conscious state; TBI subset | anodal tDCS over F3, 2 mA for 20 min, 10 sessions | sham tDCS | CRS-R; consciousness outcome | R09 | 1 | single record; no duplicate detected | DOC/consciousness outcome; TBI subset only |
| UPS036 | subacute TBI patients | left DLPFC anodal tDCS | sham tDCS | attention; executive function; memory; social perception | R01; R02; R05; R06; R09 | 5 | probable duplicate: same first author/year and highly similar title | RCT; pre-post improvements but no significant between-group difference; possible duplicate across R06-R10 reviews; possible duplicate with R01-R05 (R01, R02, R05) \| possible duplicate across reviews \| tDCS/TBI RCT; cognitive outcomes reported; possible duplicate across R06-R10 reviews; possible duplicate with R01-R05 (R01, R02, R05); merged 5 review-level rows into one unique primary study |
| UPS037 | TBI patients | rTMS + cognitive training | cognitive training | RBMT | R04 | 1 | single record; no duplicate detected | NR |
| UPS038 | chronic recurrent mild TBI subset | anodal tDCS over C3, 1 mA for 20 min, cross-over | sham tDCS | MRS GABA, TMS, PCS, cognitive assessment | R09 | 1 | single record; no duplicate detected | mixed biomarker/cognition outcome |
| UPS039 | TBI patients | rTMS + cognitive training | sham rTMS + cognitive training | MMSE; LOTCA; P300 | R04 | 1 | single record; no duplicate detected | NR |
| UPS040 | craniocerebral injury patients | rTMS + cognitive/rehabilitation training | cognitive training/control | MMSE | R04 | 1 | single record; no duplicate detected | NR |
| UPS041 | TBI patients | rTMS + cognitive training | sham rTMS + cognitive training | MoCA; LOTCA; P300; adverse reactions | R04 | 1 | single record; no duplicate detected | NR |
| UPS042 | traumatic head injury cognitive impairment patients | rTMS + cognitive training | cognitive training | MoCA; MMSE; MBI | R04 | 1 | single record; no duplicate detected | NR |
