# Supplementary material for: Non-invasive brain stimulation for cognitive impairment after traumatic brain injury: an umbrella review of systematic reviews
Source: Front Neurol. 2026 Aug 19;17:1931683. doi: 10.3389/fneur.2026.1931683 (PMC13533710; doi:10.3389/fneur.2026.1931683)
Supplement: Supplementary file 5 [file Table_5.DOCX]

Supplementary Table S5. Review-level evidence and safety reporting summary.

This table summarizes review-level evidence classifications already reported in the manuscript. No new quantitative synthesis or primary-study reanalysis was performed.

| Author, year | Type | NIBS | Population | Studies / participants | Cognitive outcomes | Review-level interpretation | Set |
| --- | --- | --- | --- | --- | --- | --- | --- |
| Ahorsu 2021 | SR + MA | Mixed NIBS | TBI; severity not restricted | 9; 197 | overall/global cognition; attention; memory; executive function | Reported estimates suggested possible benefit, but confidence in the evidence remained limited by sample size, heterogeneity, and methodological concerns. | Core |
| Alashram 2023 | SR | rTMS/tDCS | Mixed TBI severity/stage | 10; 228 | Global cognition; memory; attention; EF | Narrative evidence was limited and heterogeneous. | Core |
| Aleid 2025 | SR + MA of RCTs | rTMS | TBI; severity NR | 7; 184 | Cognitive function | Reported estimates suggested possible benefit, but confidence in the evidence remained limited by sparse domain detail and methodological concerns. | Core |
| Chen 2024 | SR + MA | rTMS + cognitive training | Post-TBI cognitive disorder | 14; 820 | MoCA; MMSE; memory; attention; P300 | Reported improvement signals for combined rTMS + cognitive training, although interpretation was limited by heterogeneity and methodological concerns; evidence did not isolate rTMS alone. | Core |
| Nousia 2022 | SR | NIBS +/- cognitive training | Mixed mild/moderate/severe TBI | 10; 232 | Attention; EF; speed; WM; memory | No clear support for NIBS or NIBS-CT improving cognition. | Core |
| Hara 2021 | SR | rTMS/tDCS | TBI rehabilitation patients | 5; 135 | Attention; memory; WM; EF | Preliminary mixed findings; insufficient evidence for routine effectiveness. | Core |
| Tsai 2021 | SR + MA | rTMS | TBI with depression/headache/PCS | 7; 136 | Visuospatial memory; speed; attention | Reported improvement signals for visuospatial memory, although interpretation was limited; other cognitive domains were not consistently demonstrated. | Core |
| Kawata 2024 | SR of RCTs | Stimulation/NIBS findings only | Adults with chronic TBI; broad review | 44 RCTs overall; participants NR | Cognition; WM; speed; fatigue | Broad review; stimulation/NIBS cognition findings only. | Supplementary |
| Zaninotto 2019 | SR | tDCS | Adult TBI; mixed recovery outcomes | 14; NR | Cognition; neuropsychology; DOC/motor | Broad tDCS review with mixed cognition, motor, and DOC outcomes. | Supplementary |
| Oktaviani 2026 | MA + TSA | rTMS | TBI; pain/neuropsychiatric focus | 9; NR | Cognitive function; PCS; depression; pain | Supplementary/sensitivity evidence; cognition secondary. | Supplementary |

Safety reporting summary.

Adverse-event reporting was limited and inconsistently presented across the included review records. Reported adverse events in individual reviews, when available, included headache, scalp discomfort, and treatment discontinuation; however, modality-specific safety reporting was insufficient across the included reviews. Some reviews focused primarily on cognitive outcomes and did not provide detailed, outcome-level safety summaries for the TBI cognitive rehabilitation population. Consequently, the available review-level evidence was insufficient to draw firm conclusions about tolerability or comparative safety. Incomplete reporting of adverse events should not be interpreted as confirming safety.
